# Sorbicillasins A–B and Scirpyrone K from a Deep-Sea-Derived Fungus, *Phialocephala* sp. FL30r

**DOI:** 10.3390/md16070245

**Published:** 2018-07-23

**Authors:** Zhenzhen Zhang, Xueqian He, Qian Che, Guojian Zhang, Tianjiao Zhu, Qianqun Gu, Dehai Li

**Affiliations:** 1 Key Laboratory of Marine Drugs, Chinese Ministry of Education, School of Medicine and Pharmacy, Ocean University of China, Qingdao 266003, China; 15192768701@163.com (Z.Z.); h19491001@163.com (X.H.); cheqian064@ouc.edu.cn (Q.C.); zhutj@ouc.edu.cn (T.Z.); guqianq@ouc.edu.cn (Q.G.); 2 Laboratory for Marine Drugs and Bioproducts of Qingdao National Laboratory for Marine Science and Technology, Qingdao 266237, China

**Keywords:** deep-sea derived fungus, *Phialocephala* sp., nitrogen-containing sorbicillinoids, radical scavenging activity

## Abstract

Two new nitrogen-containing sorbicillinoids named sorbicillasins A and B (**1** and **2**) and a new 3,4,6-trisubstituted *α*-pyrone derivative, scirpyrone K (**3**), together with two known biosynthetically related polyketides (**4**–**5**), were isolated from the deep-sea-derived fungus *Phialocephala* sp. FL30r by using the OSMAC (one strain-many compounds) method. The structures of **1**–**3**, including absolute configurations, were deduced based on MS, NMR, and time-dependent density functional theory (TD-DFT) calculations of specific ECD (electronic circular dichroism) spectra. Compounds **1** and **2** possessed a novel hexahydropyrimido[2,1-*a*] isoindole moiety, and compound **3** exhibited weak radical scavenging activity against DPPH (2,2-diphenyl-1-picrylhydrazyl) with an IC_50_ value of 27.9 μM.

## 1. Introduction

Filamentous fungi are known as prolific microbial factories for the production of a wide range of metabolites having extensive biological activity [1]. However, previous genomic analysis of fungi revealed a large number of biosynthetic genes that were unexpressed under common laboratory culture conditions, which offers a great opportunity for natural product discovery research [2]. To induce the expression of silent biogenetic clusters and increase the chemical diversity of the secondary metabolome, the approach “one strain-many compounds (OSMAC)” has been widely and successfully practiced by altering media constituents and manipulating culture conditions [3,4,5].

During our ongoing search for bioactive secondary metabolites from deep-sea-derived fungi [6], the fungal strain *Phialocephala* sp. FL30r was found to be an extensive producer of diverse polyketides, including monomeric sorbicillinol derivatives, bisorbicillinoids, and trisorbicillinoids [7,8,9,10]. Based upon the biosynthetic capability of this strain, the OSMAC approach was employed to further enhance the structural diversity of secondary metabolites. When the fungal strain was cultured on a mannitol-based medium, the HPLC-UV profile (Figure S1) of the fungal extract differed from those generated previously from culture in a potato-based medium [7,8,9,10]. Further chemical assessment of the organic extract led to the isolation of two new nitrogen-containing sorbicillinoids named sorbicillasins A and B (**1** and **2**) and a new 3,4,6-trisubstituted *α*-pyrone derivative, scirpyrone K (**3**), together with two known biosynthetically related polyketides (**4**–**5**) [11,12]. Among them, compounds **1** and **2** are sorbicillin-asparagine hybrids possessing a unique hexahydropyrimido[2,1-*a*] isoindole tricyclic skeleton. The radical scavenging activities of the new compounds against DPPH (2,2-diphenyl-1-picrylhydrazyl) were tested, and **3** showed weak activity with an IC_50_ value of 27.9 µM. Herein, we report the details of the isolation, structure elucidation, and biological activities of compounds **1**–**5**.

## 2. Results and Discussion

**Figure 1:**
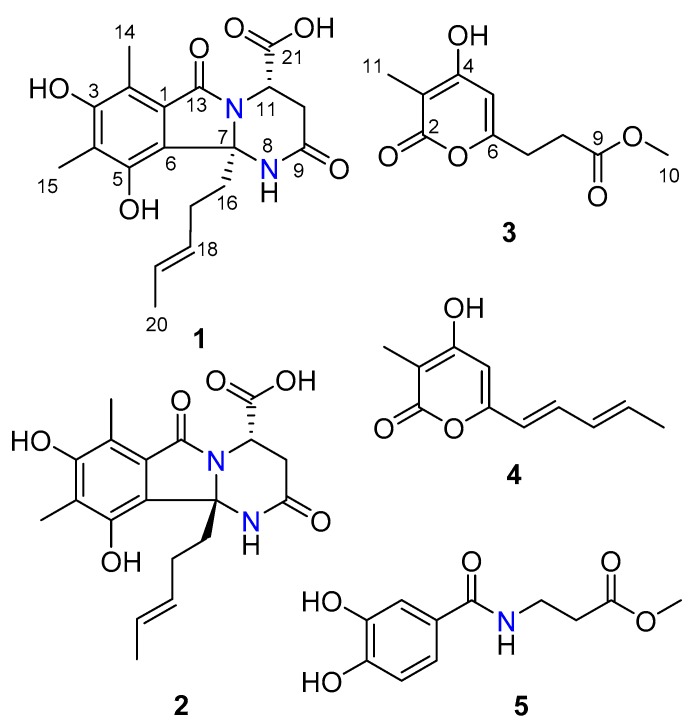
Structures of compounds **1**–**5**.

The fungus *Phialocephala* sp. FL30r was cultured in mannitol-based medium (45.0 L) with agitation. The EtOAc extract (15.0 g) of fermentation was fractionated by silica gel column chromatography, Sephadex LH-20 column chromatography, medium-pressure preparation liquid chromatography (MPLC; ODS), and semi-preparative HPLC to afford compounds **1** (4.5 mg), **2** (2.7 mg), **3** (7.0 mg), **4** (4.4 mg), and **5** (2.0 mg) (Figure 1).

**Figure 2:**
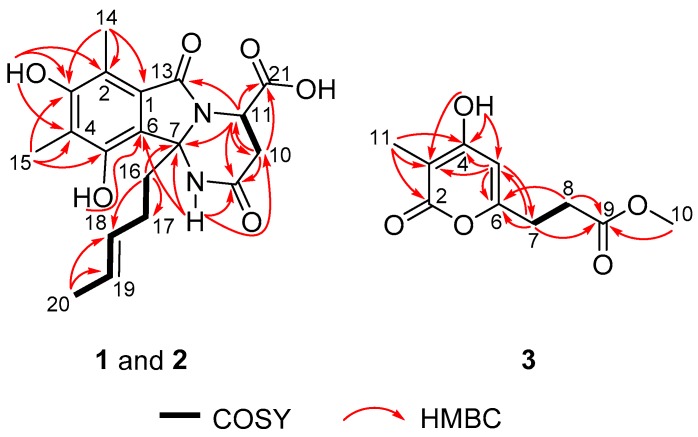
Key COSY and HMBC correlations of compounds **1**–**3**.

Sorbicillasins A and B (**1** and **2**) were both obtained as yellow oils with the molecular formula C_19_H_22_N_2_O_6_ according to the protonated peak at *m*/*z* 375.1550 (Figure S9, calcd. for C_19_H_23_N_2_O_6_, 375.1151) and the sodinated peak at *m*/*z* 397.1376 (Figure S17, calcd. for C_19_H_22_N_2_O_6_Na, 397.1370) as analyzed by HRESIMS, respectively. The 1D NMR data (Table 1) of **1** and **2** were highly similar. Each set of data suggested the presence of 19 carbons, assigned as 3 methyls, 3 methylenes, 3 methines with 2 vinyl moieties, and 10 non-protonated carbons, including 3 carbonyls. Among the three carbonyls, one was proven to be a carboxylic group based on the exchangeable proton signal at *δ*_H_ 13.12, together with the IR absorptions at 3524 and 1670 cm^−1^. The planar structures of **1** and **2** were determined to be the same by interpretation of 1D and 2D NMR spectroscopic data (Figure 2, Table 1, Figures S2–S7, and Figures S11–S15). The methylated olefinic hydrocarbon chain (from C-16 to C-20) was established by the sequential COSY correlations of H_2_-16/H_2_-17/H-18/H-19/H_3_-20. The presence of a fully substituted benzene ring was indicated by the aromatic non-protonated carbon signals (C-1/C-6) in the ^13^C-NMR spectrum. Consistent with this analysis, diagnostic HMBC correlations were observed from H_3_-14 to C-1, C-2, and C-3; from H_3_-15 to C-3, C-4, and C-5; from 3-OH to C-2, C-3, and C-4; and from 5-OH to C-6. The presence of a tetrahydro-pyrimidinone ring was postulated based on the COSY correlation (H-10/H-11) and the HMBC correlations from 8-NH to C-7, C-9, and C-10; from H_2_-10 to C-9 and C-11; and from H-11 to C-7, C-9, and C-10. The HMBC correlations from 8-NH and H_2_-16 to C-6 confirmed the connection of the tetrahydro-pyrimidinone structure and the benzene ring. The HMBC correlation from H_2_-16 to C-7 positioned the olefinic chain at C-7. Based on the key HMBC correlations from H-11 to C-13 and C-21, together with the chemical shift of C-11 (*δ*_C_ 48.2) and C-13 (*δ*_C_ 169.0), two carbonyls (C-13 and C-21) were connected to N-12 and C-11, respectively. Finally, when accounting for the molecular formula and the degree of unsaturation, C-1 was linked to C-13, and one hydroxyl group was attached to C-21, thus completing the planar structure of **1**.

**Figure 3:**
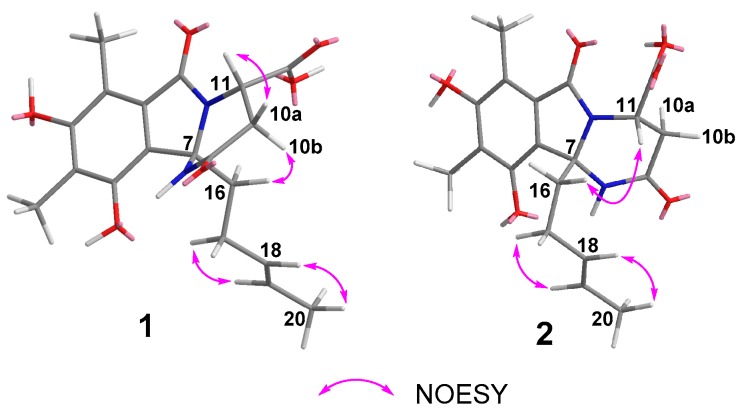
Key NOESY correlations of compounds **1** and **2**.

**Figure 4:**
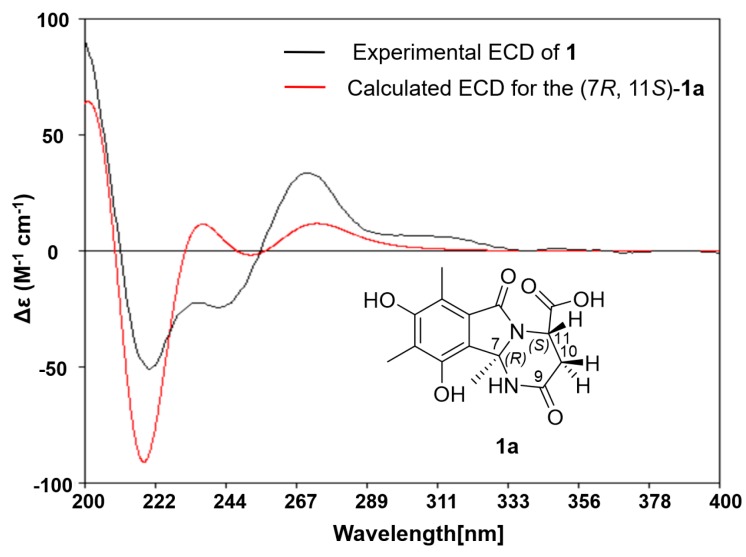
Experimental ECD spectrum of **1** (black curve) and that calculated from the truncated model **1a** (red curve) (0.30 eV).

The relative configuration of **1** was deduced based on the NOESY correlations (Figure 3 and Figure S8). The *E* geometries of double bonds in the olefinic chain were deduced by the correlations between H-17 and H-19 and between H-18 and H-20. The NOEs of H-10a/H-11 and H-10b/H-16 indicated that the carboxylic group and methylated olefinic hydrocarbon chain were to the same face of the pyrimidinone ring. Thus, the relative configuration of **1** was suggested to be 7R*, 11S*. The absolute configuration of **1** was determined by comparing the experimental ECD curve with the one calculated from the truncated model (7*R*,11*S*)-**1a** using time-dependent density functional theory (TD-DFT). The DFT re-optimization of the initial MMFF (Merck molecular force field) minima was performed at the B3LYP/6-31+g(d) level with a polarizable continuum model (PCM) solvent for MeOH. The strong agreement between the calculated ECD spectra of (7*R*,11*S*)-**1a** with experimental results suggested the absolute configuration of **1** as 7*R*, 11*S* (Figure 4 and Figure S27).

**Figure 5:**
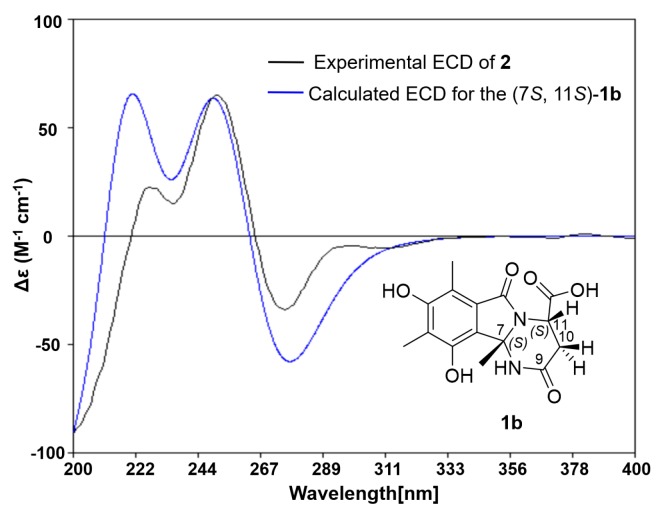
Experimental ECD spectrum of **2** (black curve) and that calculated from the truncated model **1b** (blue curve) (0.35 eV).

The slight discrepancies of **1** and **2** in the NMR data suggested they might be isomers. Further NOESY correlation (Figure 3 and Figure S16) of H-11/H_2_-16 indicated that the relative configuration of **2** was 7S*, 11S*. The absolute configuration of **2** was then further determined as 7*S*, 11*S* by the agreement between the calculated ECD spectra of **2** and the experimental results according to the truncated model (7*S*,11*S*)-**1b** (Figure 5 and Figure S28).

Compound **3** was obtained as a white amorphous powder, and the molecular formula was determined to be C_10_H_12_O_5_ by HRESIMS peaks at *m*/*z* 213.0764 (Figure S25, calcd. for C_10_H_13_O_5_, 213.0757). The 1D NMR data (Table 2) of **3** suggested the presence of two methyls, including one methoxy (*δ*_C_ 52.0 and *δ*_H_ 3.58), two methylenes, one methine, and five non-protonated carbons. Comparison of the ^1^H and ^13^C NMR spectra (Figures S19–S21) of **3** with those of scirpyrone H revealed the presence of an extra methyl group (*δ*_C_ 8.8 and *δ*_H_ 1.72) and the replacement of the 4-methoxyl group by a 4-hydroxyl group in **3** [13]. Further 2D NMR (Figures S22–S24) data and key HMBC correlations from H_3_-11 to C-2, C-3, and C-4, from H-5 to C-3, C-4, and C-6, and from 4-OH to C-3, C-4, and C-5 supported the locations of the 4-hydroxyl group and the 11-methyl group.

Compounds **4** and **5** were identified as trichopyrone [11] and peniginseng A [12] based on the comparison of their spectroscopic data (NMR and MS) with those reported in the literature.

The cytotoxicity against K562 and MGC-803 cell lines [14,15] and the radical scavenging activity [16] against DPPH of the new compounds **1**–**3** were evaluated. All of them were non-cytotoxic. Compound **3** exhibited weak activity against DPPH with an IC_50_ value of 27.9 μM (ascorbic acid was used as a positive control with an IC_50_ value of 14.2 μM), whereas compounds **1** and **2** were not active (IC_50_ > 500 μM). According to the literature [11,12], the known compound **4** showed weak radical scavenging activity, but the radical scavenging activity of **5** has not been reported.

**Figure 6:**
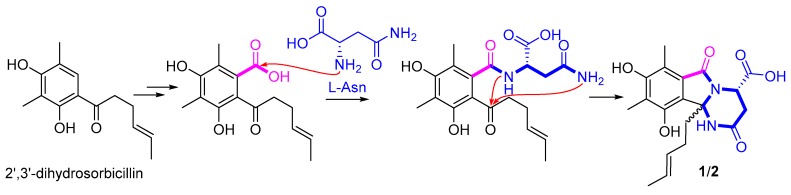
Possible biosynthetic pathway of compounds **1** and **2**.

Sorbicillinoids belong to a large family of polyketides with highly diverse carbon skeletons and bioactivities [17]. Since first reported in 1948, about 90 sorbicillinoids have been isolated from terrestrial- and marine-derived fungi [17]. Among them, the nitrogen-containing analogues are rare, with only eight related cases reported, including sorbicillactones A and B [18], sorbicillinoid urea [19], and sorbicillamines A–E [20]. The sources of nitrogen atoms in the reported nitrogenous sorbicillinoids were deduced to be l-alanine, urea, and an aminotransferase enzyme [18,19,20]. In this report, sorbicillasins A and B (**1** and **2**) were probably formed by adding a whole molecule of l-asparagine to 2′,3′-dihydrosorbicillin [21] (Figure 6) via sequential intermolecular/intramolecular nucleophilic reactions. The hexahydropyrimido[2,1-*a*] isoindole ring system in compounds **1** and **2**, which compose a 6/5/6 tricyclic ring system, have not been found in nature, with only related synthetic structures reported [22,23,24]. The above result shows that the OSMAC approach is a useful method to discover structurally diversified metabolites from a deep-sea-derived fungal strain.

## 3. Materials and Methods

### 3.1. General Experimental Procedures

UV spectra were recorded on a Beckman DU 640 spectrophotometer (Beckman Coulter Inc., Brea, CA, USA). Specific rotations were measured with a JASCO P-1020 digital polarimeter (JASCO Corporation, Tokyo, Japan). ESIMS were obtained on a Thermo Scientific LTQ Orbitrap XL mass spectrometer (Thermo Fisher Scientific, Waltham, MA, USA) or a Micromass Q-TOF ULTIMA GLOBAL GAA076 LC Mass spectrometer (Wasters Corporation, Milford, MA, USA). CD spectra were measured on a JASCO J-815 spectropolarimeter (JASCO Corporation, Tokyo, Japan). NMR spectra were recorded with an Agilent 500 MHz DD2 spectrometer (Agilent Technologies Inc., Santa Clara, CA, USA) using TMS as an internal standard, and chemical shifts were recorded as *δ*-values. Semi-preparative HPLC (Hitachi, Tokyo, Japan) was performed on an ODS column (YMC-Pack ODS-A, 10 mm × 250 mm, 5 μm, 3 mL/min, YMC. Co., Ltd., Tokyo, Japan). Medium-pressure preparation liquid chromatography (MPLC) was performed on a Bona-Agela CHEETAHTM HP100 (Beijing Agela Technologies Co., Ltd., Beijing, China). Column chromatography (CC) was performed with silica gel (200–300 mesh, Qingdao Marine Chemical Inc., Qingdao, China) and Sephadex LH-20 (Amersham Biosciences, San Francisco, CA, USA) [25].

### 3.2. Fungal Material

The fungal strain FL30r has been previously described [7,8,9,10]. The strain was deposited at the Key Laboratory of Marine Drugs, the Ministry of Education of China, School of Medicine and Pharmacy, Ocean University of China, Qingdao, China.

### 3.3. Fermentation and Extraction 

Erlenmeyer flasks (500 mL) containing 150 mL fermentation medium were directly inoculated with spores. The media contained mannitol (20.0 g), glucose (20.0 g), peptone (10.0 g), yeast extract (5.0 g), corn syrup (1.0 g), KH_2_PO_4_ (0.5 g), and MgSO_4_·7H_2_O (0.3 g) dissolved in 1 L of naturally collected seawater (Huiquan Bay, Yellow sea). The flasks were cultured at 28 °C on a rotary platform shaker at 180 rpm for 9 days. The fermentation broth (45.0 L) was filtered through cheese cloth to separate the supernatant from mycelia. The supernatant was extracted with EtOAc (3 × 45.0 L) and evaporated under reduced pressure to produce the crude gum (15.0 g) [26].

### 3.4. Isolation 

The extract was subjected to VLC (vacuum liquid chromatography) and a stepped gradient elution with petroleum ether/EtOAc (10:0 to 0:10), and EtOAc/MeOH (10:0 to 0:10) was applied to give eight fractions (Fr.1 to Fr.8). Fr.5 was further separated on a Sephadex LH-20 column eluted with MeOH and provided four subfractions (Fr.5-1 to Fr.5-4). Fr.5-3 was then separated by semi-preparative HPLC with MeOH/H_2_O (40:60) as a mobile phase to give compound **1** (4.5 mg, *t*_R_ 17 min). Fr.6 was further separated by MPLC (C-18 ODS) using a stepped gradient elution with MeOH/H_2_O (20:80 to 100:0) to yield five subfractions (Fr.6-1 to Fr.6-5). Fr.6-1 was separated on a Sephadex LH-20 column eluted with MeOH to provide four subfractions (Fr.6-1-1 to Fr.6-1-4). Fr.6-1-3 was further separated by MPLC (C-18 ODS) using a stepped gradient elution with MeOH/H_2_O (30:70 to 100:0) to furnish nine subfractions (Fr.6-1-3-1 to Fr.6-1-3-9). Fr.6-1-3-5 was further separated by semi-preparative HPLC eluted with MeOH/H_2_O (30:70) to provide compound **3** (7.0 mg *t*_R_ 16 min). Fr.6-1-3-9 was further purified by semi-preparative HPLC eluted with MeCN/H_2_O (25:75) to give compound **2** (2.7 mg, *t*_R_ 16 min). Fr.6-1-4 was further separated by semi-preparative HPLC eluted with MeOH/H_2_O (60:40), thus providing compounds **4** (4.4 mg, *t*_R_ 13 min) and **5** (2.0 mg, *t*_R_ 35 min).

Sorbicillasin A (**1**): pale yellow oil, ${\left[\alpha \right]}_{D}^{25}$ −15.5 (c 0.15, MeOH); UV (MeOH) *λ*_max_ (log *ε*): 220 (2.65), 261 (2.01), 307 (1.80) nm; IR (KBr) *ν*_max_ 3292, 2906, 1670, 1457, 1362, 1207, 1146, 899 cm^−1^, see Figure S10; ECD (1 mM MeOH) *λ*_max_ (Δ*ε*) 220 (−3.04), 245 (−1.76), 270 (+2.32) nm; 1D NMR data, see Table 1; HRESIMS *m*/*z* 375.1550 [M + H]^+^ (calcd for C_19_H_23_N_2_O_6_, 375.1551).

Sorbicillasin B (**2**): pale yellow oil, ${\left[\alpha \right]}_{D}^{25}$ +10.4 (c 0.15, MeOH); UV (MeOH) *λ*_max_ (log *ε*): 220 (2.65), 261 (2.01), 307 (1.80) nm; IR (KBr) *ν*_max_ 3329, 2926, 1683, 1558, 1384, 1208, 1143, 838 cm^−1^, see Figure S18; ECD (1 mM MeOH) *λ*_max_ (Δ_ε_) 225 (+1.88), 250 (+4.32), 275 (−1.90) nm; 1D NMR data, see Table 1; HRESIMS *m*/*z* 397.1376 [M + Na]^+^ (calcd for C_19_H_22_N_2_O_6_Na, 397.1370).

Scirpyrone K (**3**): white amorphous powder; UV (MeOH) *λ*_max_ (log *ε*): 231 (1.36), 285 (2.35) nm; IR (KBr) *ν*_max_ 3104, 2954, 2705, 1743, 1407, 1170, 1001, 836 cm^−1^, see Figure S26; 1D NMR data, see Table 2; HRESIMS *m*/*z* 213.0764 [M + H]^+^ (calcd for C_10_H_13_O_5_, 213.0757).

### 3.5. Biological Assay

Cytotoxic activities of **1**–**3** were evaluated using an MGC-803 cell line by the SRB (Sulforhodamine B) method [14] and the K562 cell line by the MTT method [15]. The positive control was doxorubicin hydrochloride. In the DPPH scavenging assay [16], samples to be tested were dissolved in MeOH and the solution (160 μL) was dispensed into wells of a 96-well microtiter tray. Forty microliters of the DPPH solution in MeOH were added to each well. The mixture was shaken and left to stand for 30 min. After the reaction, absorbance was measured at 510 nm, and the percent inhibition was calculated. IC_50_ values denoted the concentration of sample required to scavenge 50% of the DPPH free radicals [27].

### 3.6. Computation Section

Conformational searches were run by employing the “systematic” procedure implemented in *Spartan′14* [28] using MMFF (Merck molecular force field), which were all reoptimized with DFT calculations at the B3LYP/6-31+G(d) level using the Gaussian09 program [29]. The geometry of various initial conformations was optimized. Vibrational frequency calculations confirmed the presence of minima. Time-dependent DFT calculations were performed on the three lowest-energy conformations for (7*R*,11*S*)-**1a** and three lowest-energy conformations for (7*S*,11*S*)-**1b** (>4% population) using 30 excited states and a polarizable continuum model (PCM) for MeOH. ECD spectra were generated using the program *SpecDis* [30] by applying a Gaussian band shape with 0.30 eV width for **1a** and 0.35 eV width for **1b**, from dipole length rotational strengths. The dipole velocity forms yielded negligible differences. The spectra of the conformers were combined using Boltzmann weighting, with the lowest-energy conformations accounting for 100% of the weights. The calculated spectra were shifted for **1a** (4 nm) and for **1b** (0 nm) to facilitate comparison to the experimental data.

## 4. Conclusions

In summary, two new nitrogen-containing sorbicillinoids, one new 3,4,6-trisubstituted α-pyrone derivative, and two known biosynthetically related polyketides were isolated from the deep-sea-derived fungus *Phialocephala* sp. FL30r. The absolute configurations of new compounds **1**–**2** were determined by NMR and TD-DFT calculations of specific ECD spectra. Compounds **1** and **2** were unusual naturally occurring nitrogen-containing sorbicillinoid derivatives with a novel hexahydropyrimido[2,1-*a*] isoindole moiety. Pyrone **3** exhibited radical scavenging activity against DPPH.

## Figures and Tables

**Table 1:** ^1^H (500 MHz) and ^13^C (125 MHz) NMR data of compounds **1** and **2** (DMSO-*d*_6_, *δ* ppm).

No.	1		2	
	*δ*_C_	*δ*_H_, Mult. (*J* in Hz)	*δ*_C_	*δ*_H_, Mult. (*J* in Hz)
1	125.6		125.6	
2	115.6		115.8	
3	155.7		155.8	
4	118.9		119.2	
5	148.4		148.4	
6	124.8		130.7	
7	75.2		75.2	
9	167.1		168.0	
10	31.0	2.48 dd (17.1, 6.0), H-10a 2.82 dd (17.1, 6.0), H-10b	34.0	2.35 m, H-10a 2.61 m, H-10b
11	48.2	4.94 t (7.55)	50.2	4.31 s
13	169.0		169.5	
14	10.1	2.38 s	10.2	2.33 s
15	10.7	2.11 s	10.7	2.10 s
16	36.9	2.41 dt (11.8, 4.0) 1.90 dt (11.8, 4.0)	35.9	2.45 m 2.13 m
17	27.1	1.80 dt (11.8, 5.8) 1.12 dt (11.8, 5.8)	27.3	1.66 m 1.32 m
18	130.4	5.19 m	130.5	5.24 m
19	124.8	5.17m	125.1	5.19 m
20	18.2	1.48 d (4.2)	18.3	1.48 d (5.7)
21	172.6		171.4	
3-OH		8.62 s		8.60 s
5-OH		8.77 s		8.76 s
8-NH		8.04 s		8.03 s
21-OH		13.12 brs		12.74 brs

**Table 2:** ^1^H (500 MHz) and ^13^C (125 MHz) NMR data of compound **3** (DMSO-*d*_6_, *δ* ppm).

No.	3 *^a^*	
	*δ*_C_	*δ*_H_, Mult. (*J* in Hz)
2	165.3	
3	97.3	
4	165.2	
5	99.9	5.98 s
6	161.2	
7	28.4	2.67 t (6.95)
8	30.2	2.59 t (6.95)
9	172.4	
10	52.0	3.58 s
11	8.8	1.72 s
4-OH		11.15 brs

*^a^* Recorded in DMSO.

## Supplementary Materials

The following are available online at http://www.mdpi.com/1660-3397/16/7/245/s1: the HPLC analysis of the metabolic extracts, MS, IR, 1D, and 2D NMR spectra for compounds **1**–**3**, and details for ECD calculations. Figure S1: HPLC analysis of the metabolic extracts from different conditions; Figures S2–S10: 1D, 2D NMR, NOESY, HRESIMS, and IR spectra of sorbicillasin A (**1**); Figures S11–S18: 1D, 2D NMR, NOESY, HRESIMS, and IR spectra of sorbicillasin B (**2**); Figures S19–S26: 1D, 2D NMR, HRESIMS, and IR spectra of scirpyrone K (**3**); Figure S27: ECD calculation of **1a**; Figure S28: ECD calculation of **1b**.
