# Supplementary material for: Sorbicillasins A–B and Scirpyrone K from a Deep-Sea-Derived Fungus, Phialocephala sp. FL30r
Source: Mar Drugs. 2018 Jul 23;16(7):245. doi: 10.3390/md16070245 (PMC6070939; doi:10.3390/md16070245)
Supplement: Supplementary file 1 [file marinedrugs-16-00245-s001.pdf]

Supporting Information

Sorbicillasins A–B and Scirpyrone K from a Deep-Sea-Derived Fungus, *Phialocephala* sp. FL30r

Zhenzhen Zhang,[1] Xueqian He,[1] Qian Che,[1] Guojian Zhang,[1,2,] Tianjiao Zhu,[1] Qianqun Gu,[1] and Dehai Li[1,2,*]*

[1] Key Laboratory of Marine Drugs, Chinese Ministry of Education, School of Medicine and Pharmacy, Ocean University, Qingdao 266003, P. R. China

[2] Laboratory for Marine Drugs and Bioproducts of Qingdao National Laboratory for Marine Science and Technology, Qingdao, 266237, P. R. China

List of supporting information

Figure S1. HPLC analysis of the metabolic extracts of different conditions. 3
Figure S2. ^{1}H NMR (500 MHz, DMSO-d_6) spectrum of **1**. 4
Figure S3. ^{13}C NMR (125 MHz, DMSO-d_6) spectrum of **1**. 4
Figure S4. DEPT (125 MHz, DMSO-d_6) spectrum of **1**. 5
Figure S5. HSQC spectrum of **1** in DMSO-d_6. 5
Figure S6. ^{1}H-^{1}H COSY spectrum of **1** in DMSO-d_6. 6
Figure S7. HMBC spectrum of **1** in DMSO-d_6. 6
Figure S8. NOESY spectrum of **1** in DMSO-d_6. 7
Figure S9. HRESIMS spectrum of **1**. 7
Figure S10. IR spectrum of **1**. 8
Figure S11. ^{1}H NMR (500 MHz, DMSO-d_6) spectrum of **2**. 8
Figure S12. ^{13}C NMR (125 MHz, DMSO-d_6) spectrum of **2**. 9
Figure S13. HSQC spectrum of **2** in DMSO-d_6. 9
Figure S14. ^{1}H-^{1}H COSY spectrum of **2** in DMSO-d_6. 10
Figure S15. HMBC spectrum of **2** in DMSO-d_6. 10
Figure S16. NOESY spectrum of **2** in DMSO-d_6. 11
Figure S17. HRESIMS spectrum of **2**. 11
Figure S18. IR spectrum of **2**. 12
Figure S19. ^{1}H NMR (500 MHz, DMSO-d_6) spectrum of **3**. 12
Figure S20. ^{13}C NMR (125 MHz, DMSO-d_6) spectrum of **3**. 13
Figure S21. DEPT (125 MHz, DMSO-d_6) spectrum of **3**. 13
Figure S22. HSQC spectrum of **3** in DMSO-d_6. 14
Figure S23. ^{1}H-^{1}H COSY spectrum of **3** in DMSO-d_6. 14
Figure S24. HMBC spectrum of **3** in DMSO-d_6. 15
Figure S25. HRESIMS spectrum of **3**. 15
Figure S26. IR spectrum of **3**. 16
Figure S27. ECD calculation of **1a.** 16
Figure S28. ECD calculation of **1b**. 20

Figure S1. HPLC analysis of the metabolic extracts of different conditions.

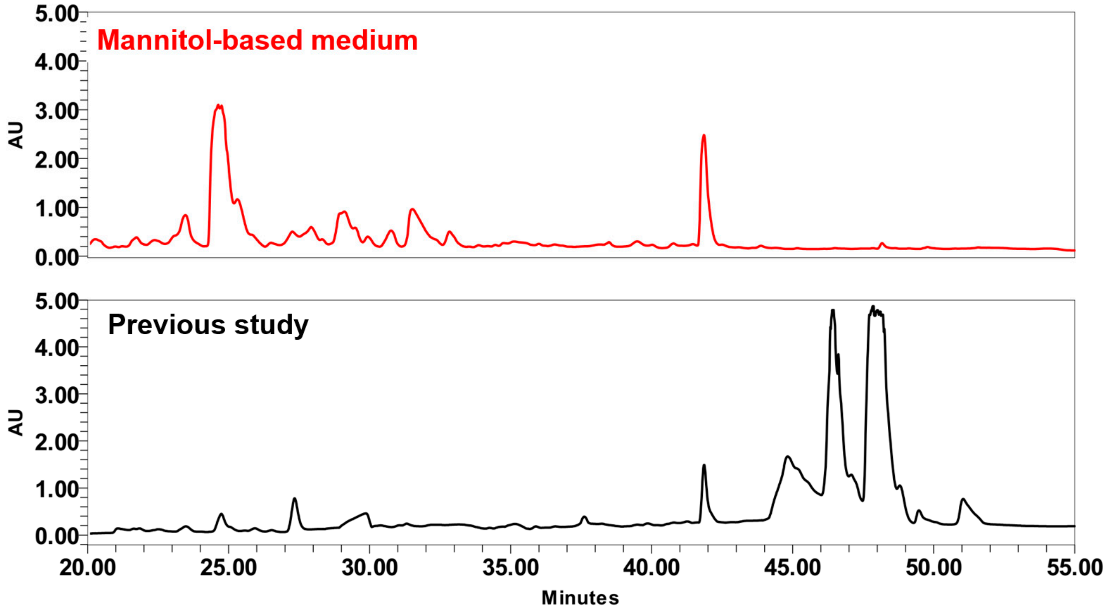

Figure S2. ^{1}H NMR (500 MHz, DMSO-d_6) spectrum of **1**.

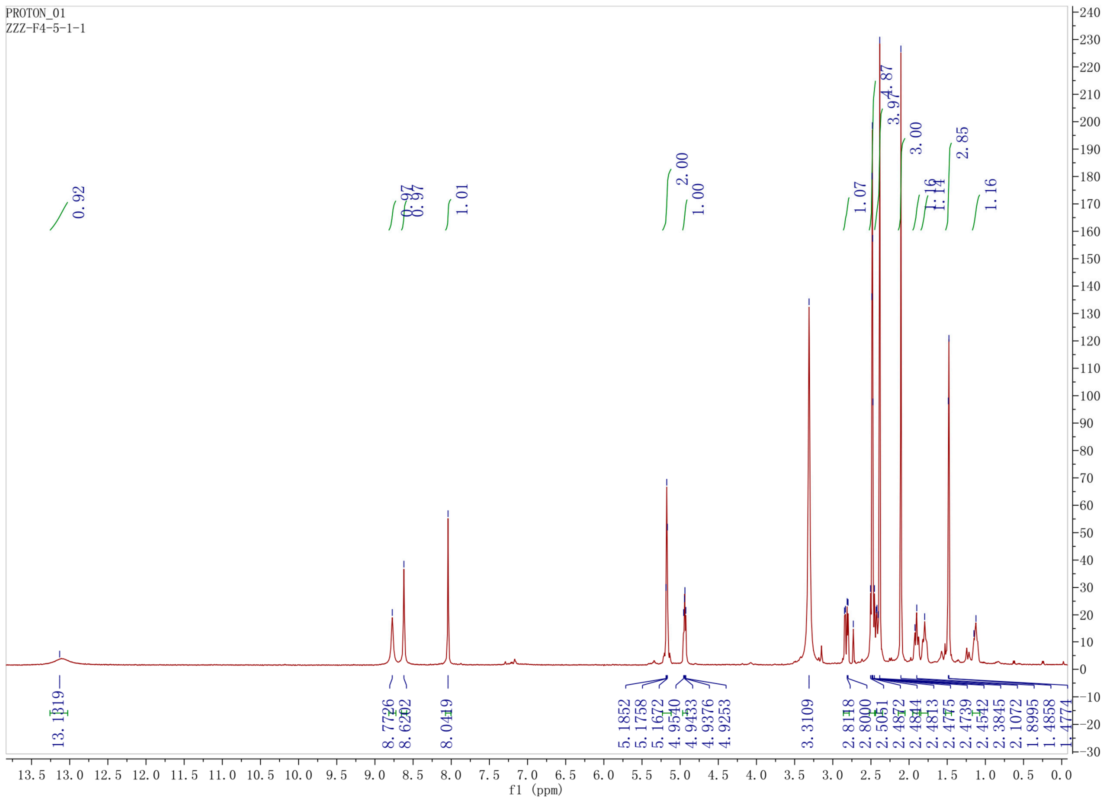

Figure S3. ^{13}C NMR (125 MHz, DMSO-d_6) spectrum of **1**.

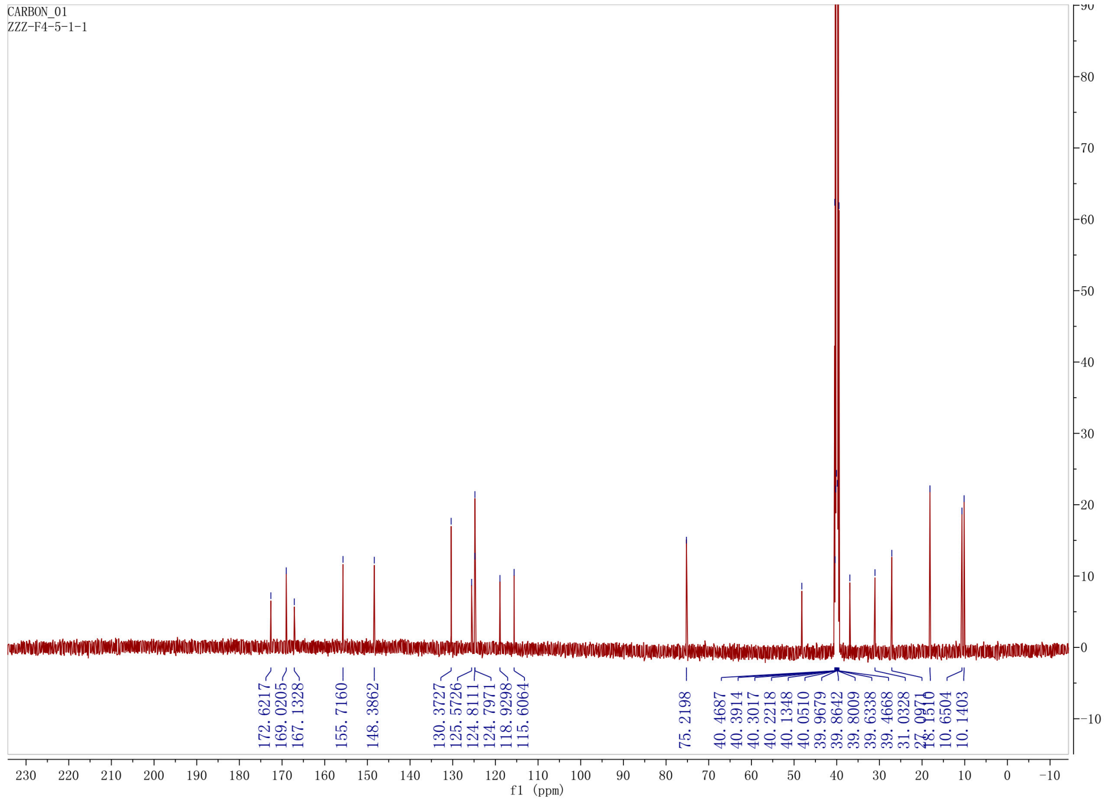

Figure S4. DEPT (125 MHz, DMSO-d_6) spectrum of **1**.

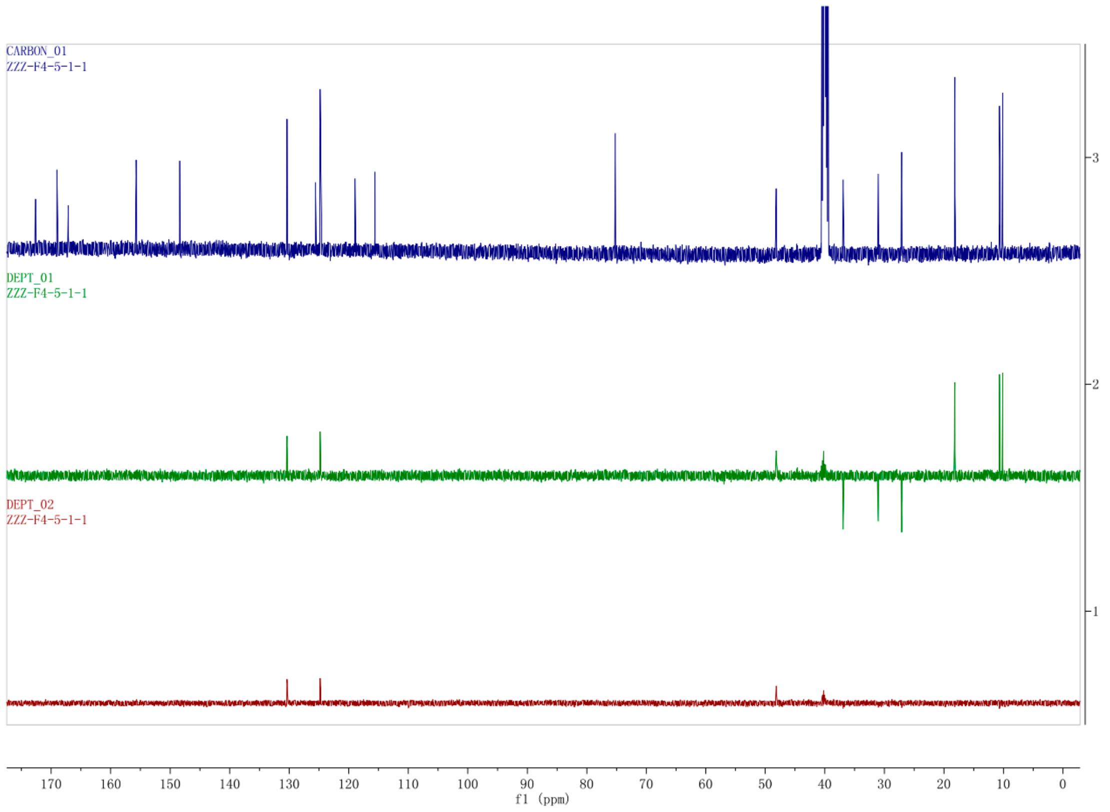

Figure S5. HSQC spectrum of **1** in DMSO-d_6.

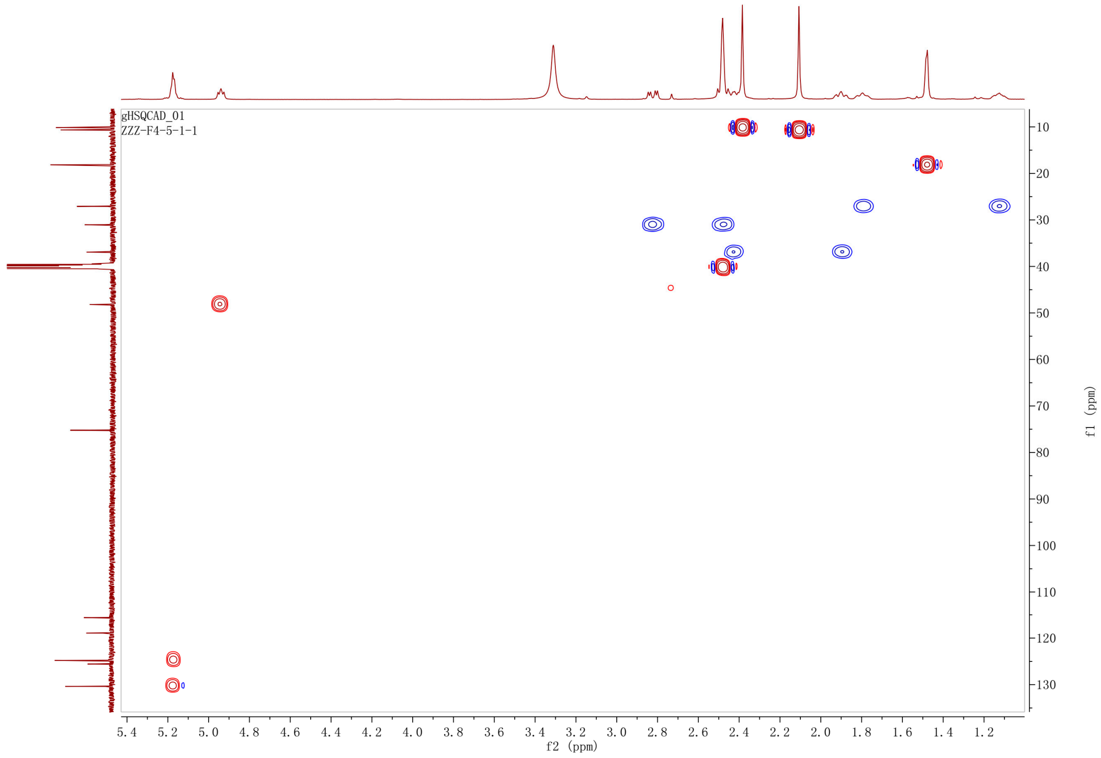

Figure S6. ^{1}H-^{1}H COSY spectrum of **1** in DMSO-d_6.

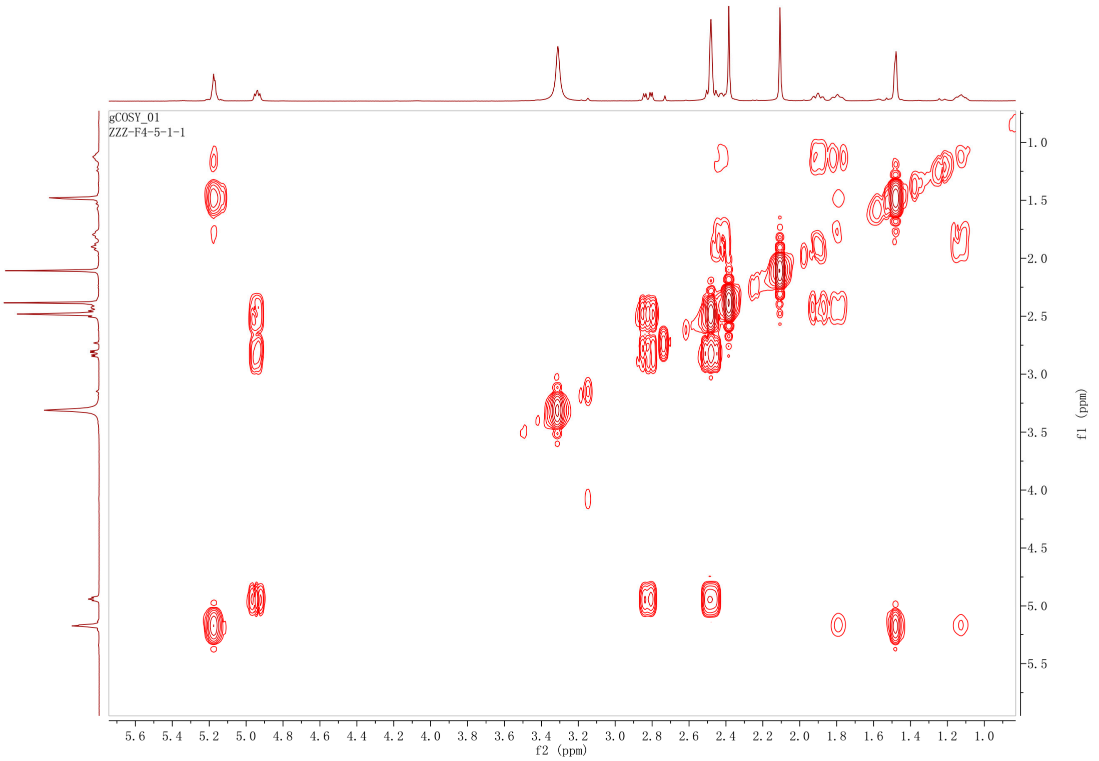

Figure S7. HMBC spectrum of **1** in DMSO-d_6.

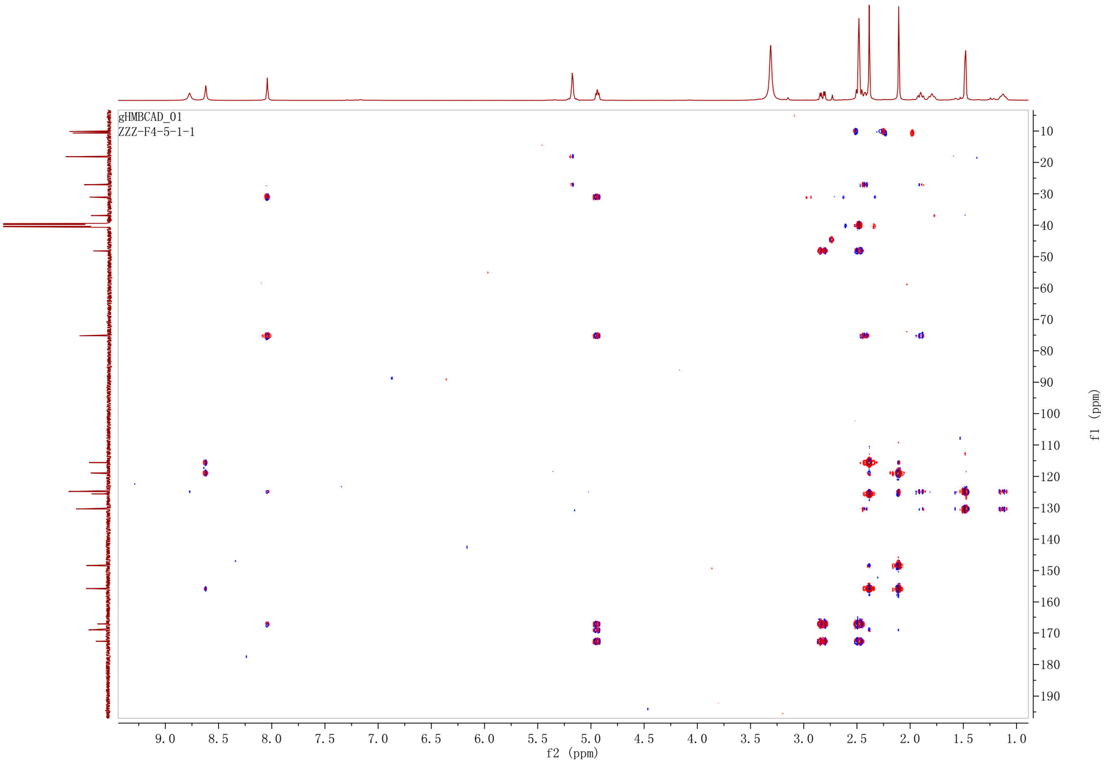

Figure S8. NOESY spectrum of **1** in DMSO-d_6.

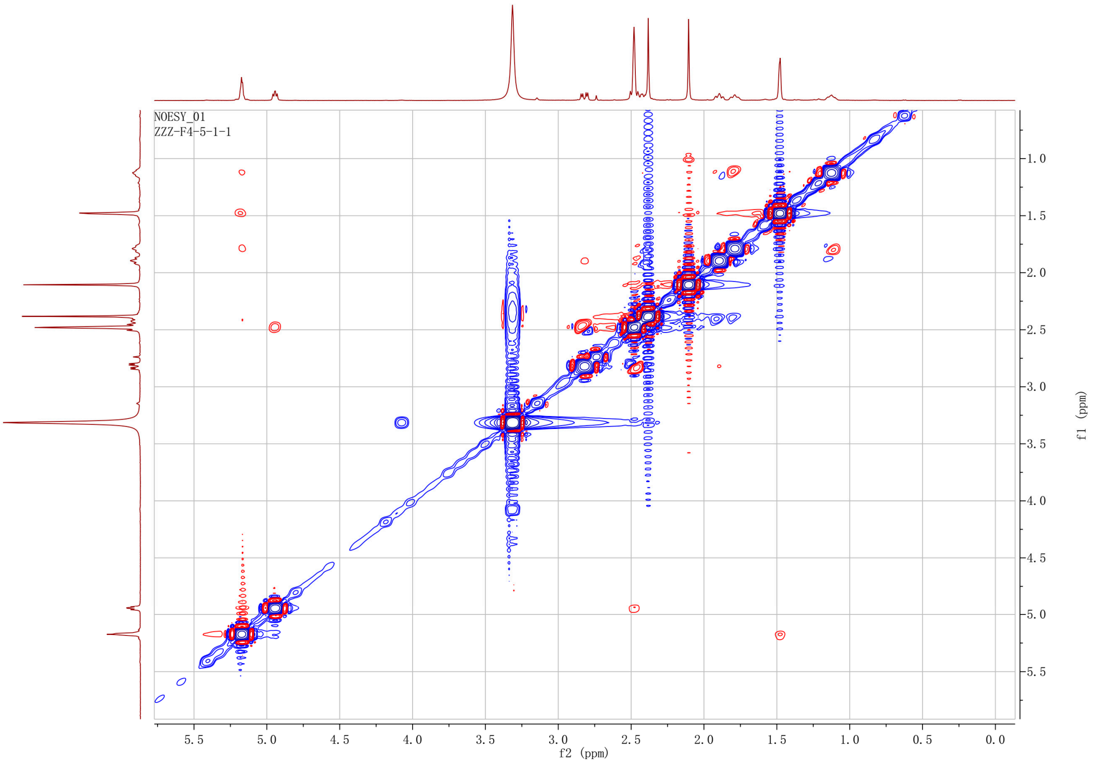

Figure S9. HRESIMS spectrum of **1**.

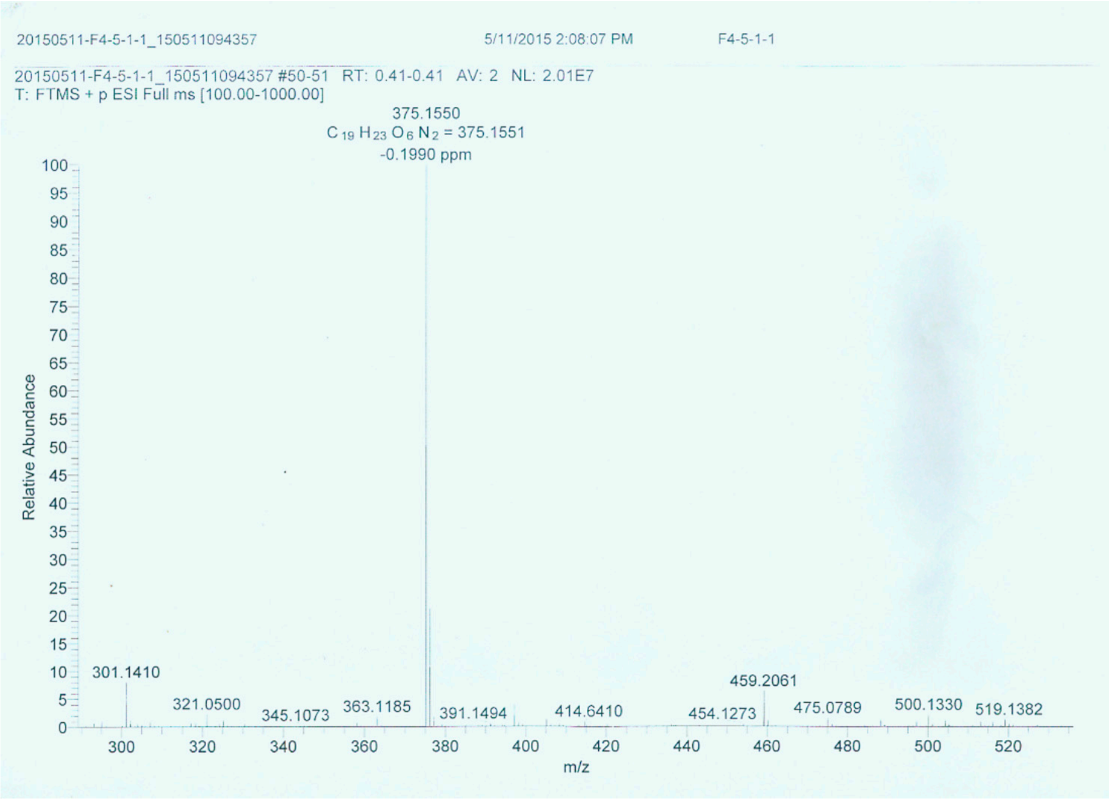

Figure S10. IR spectrum of **1**.

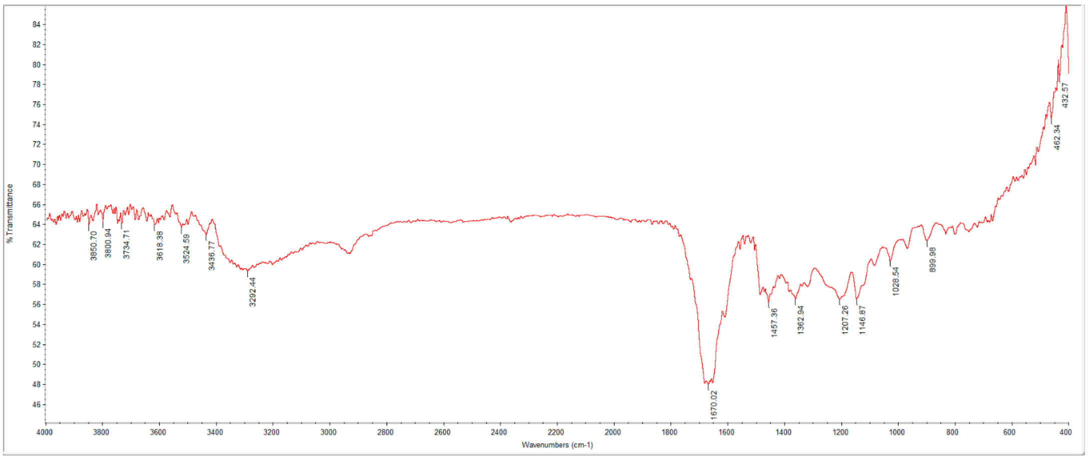

Figure S11. ^{1}H NMR (500 MHz, DMSO-d_6) spectrum of **2**.

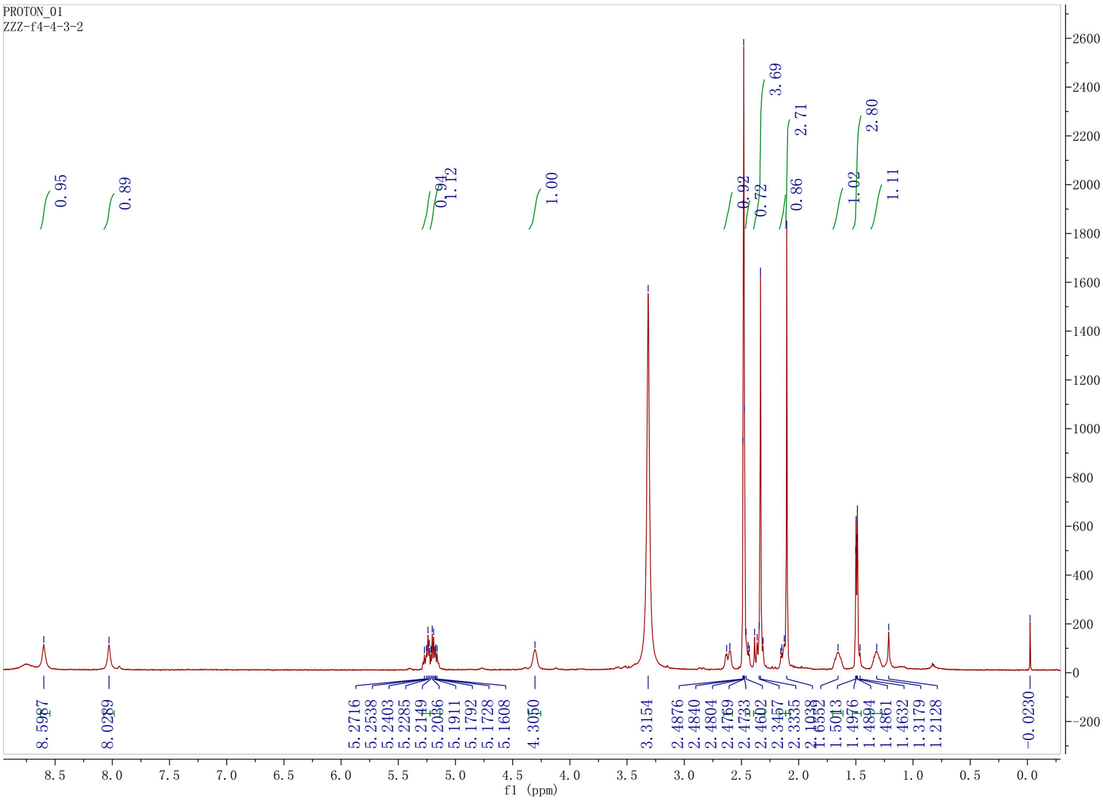

Figure S12. ^{13}C NMR (125 MHz, DMSO-d_6) spectrum of **2**.

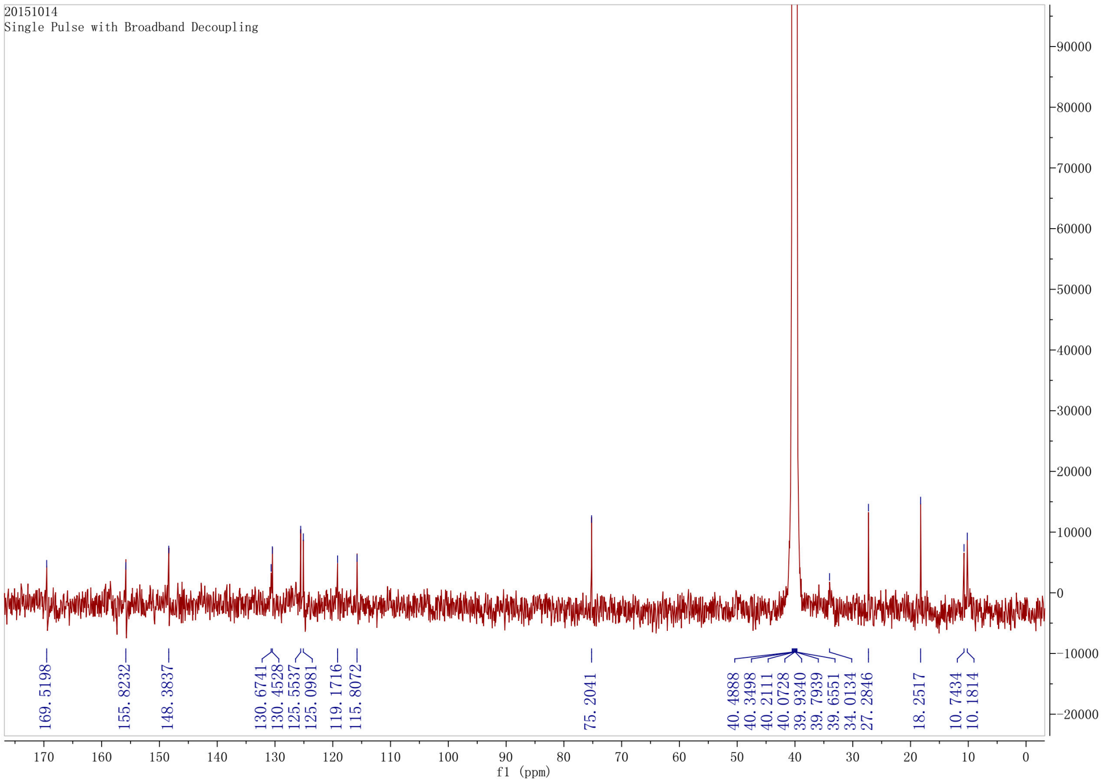

Figure S13. HSQC spectrum of **2** in DMSO-d_6.

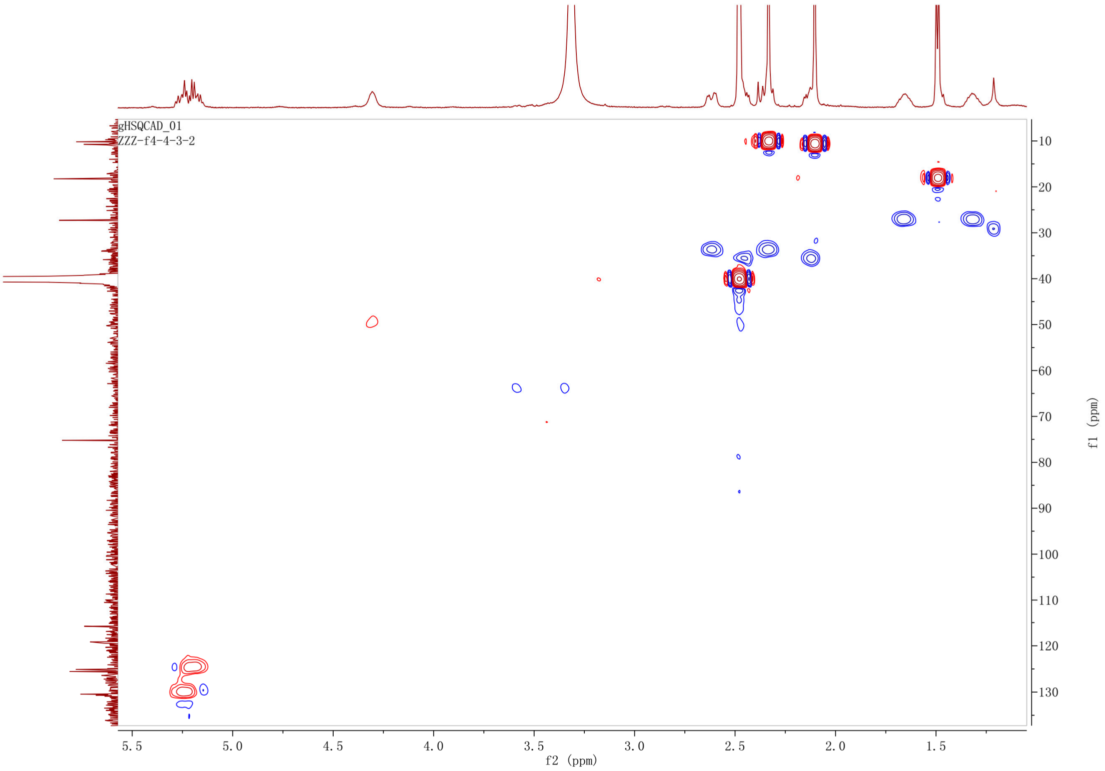

Figure S14. ^{1}H-^{1}H COSY spectrum of **2** in DMSO-*d*6.

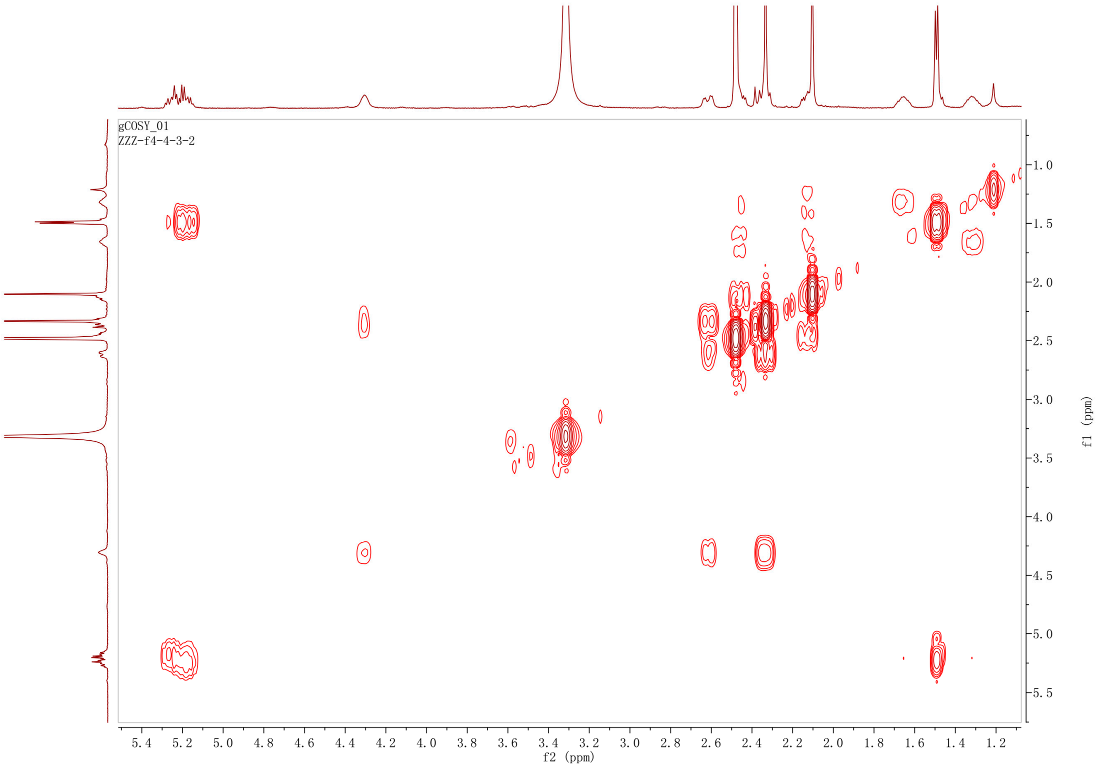

Figure S15. HMBC spectrum of **2** in DMSO-*d*6.

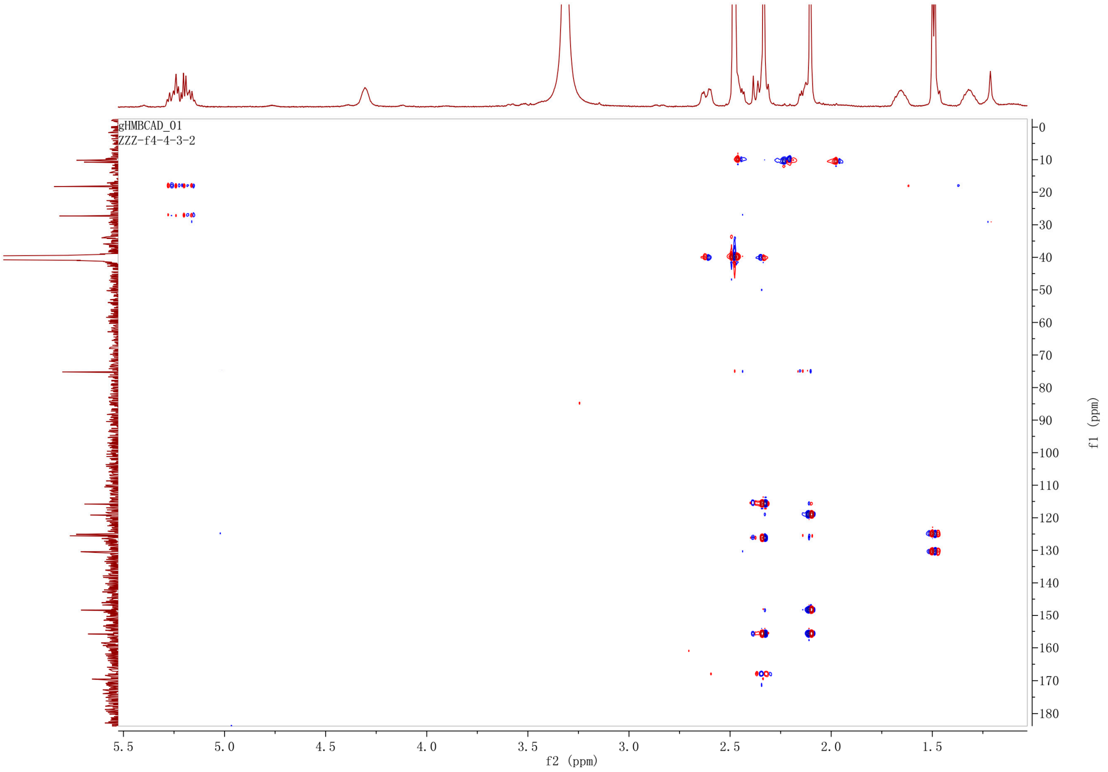

Figure S16. NOESY spectrum of **2** in DMSO-d_6.

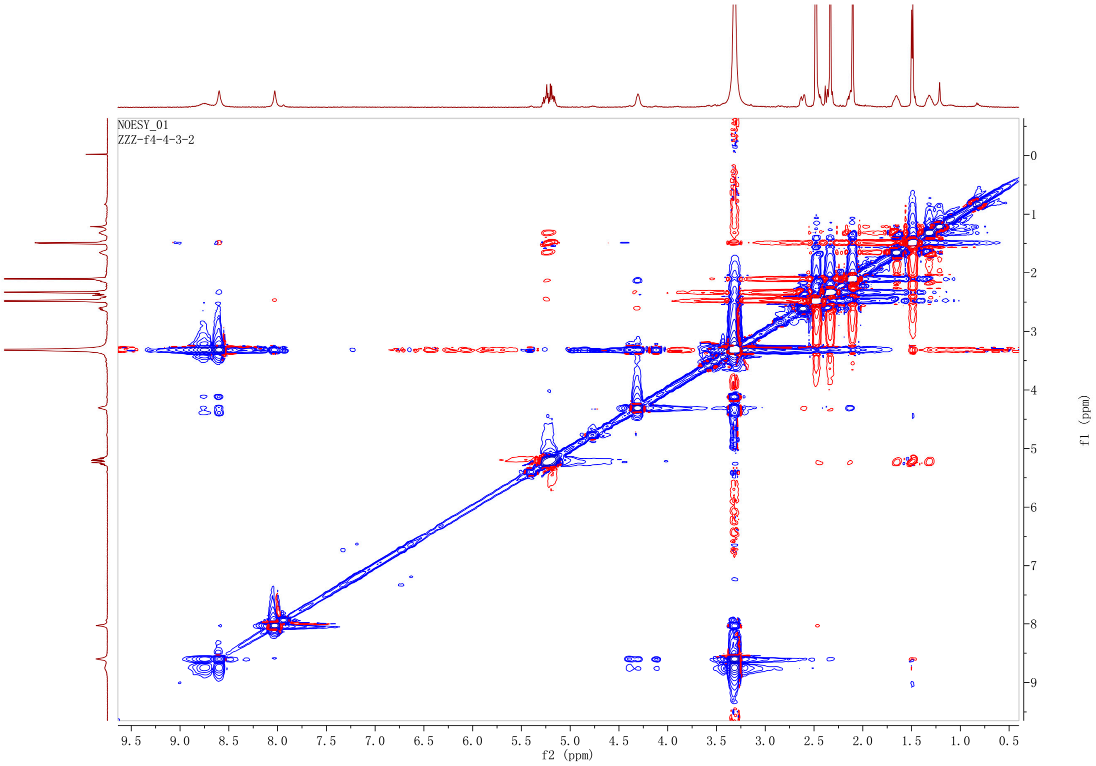

Figure S17. HRESIMS spectrum of **2**.

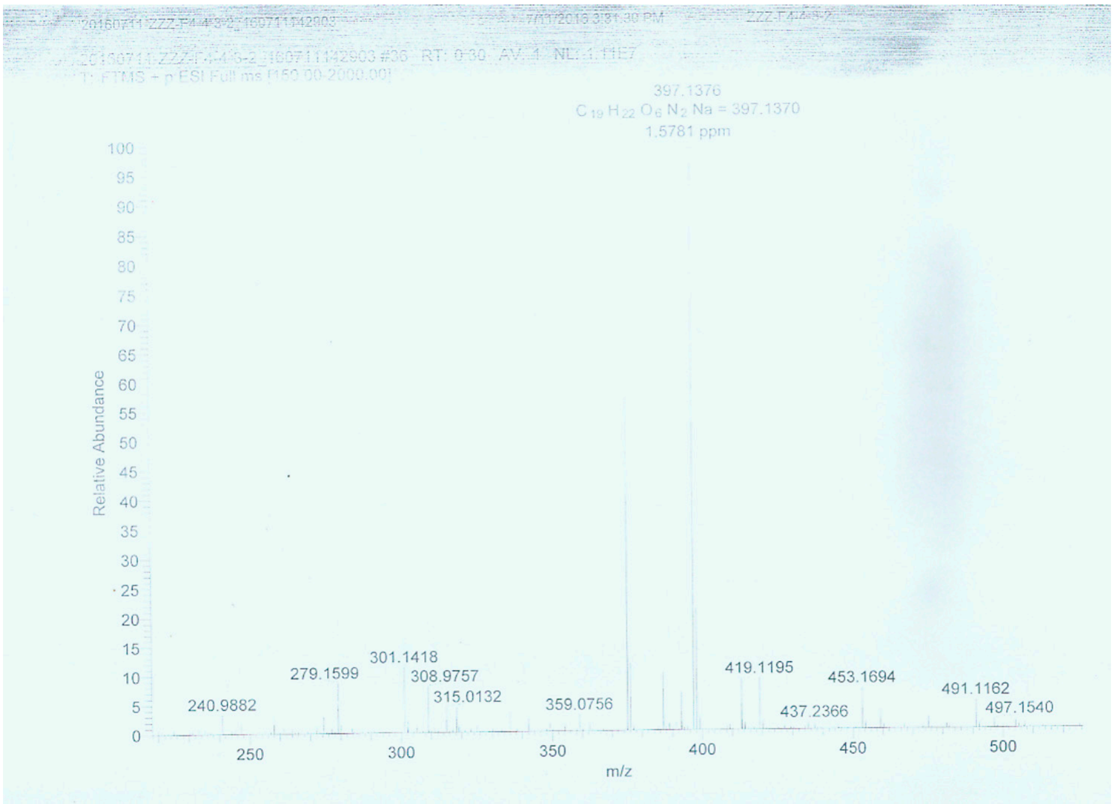

Figure S18. IR spectrum of **2**.

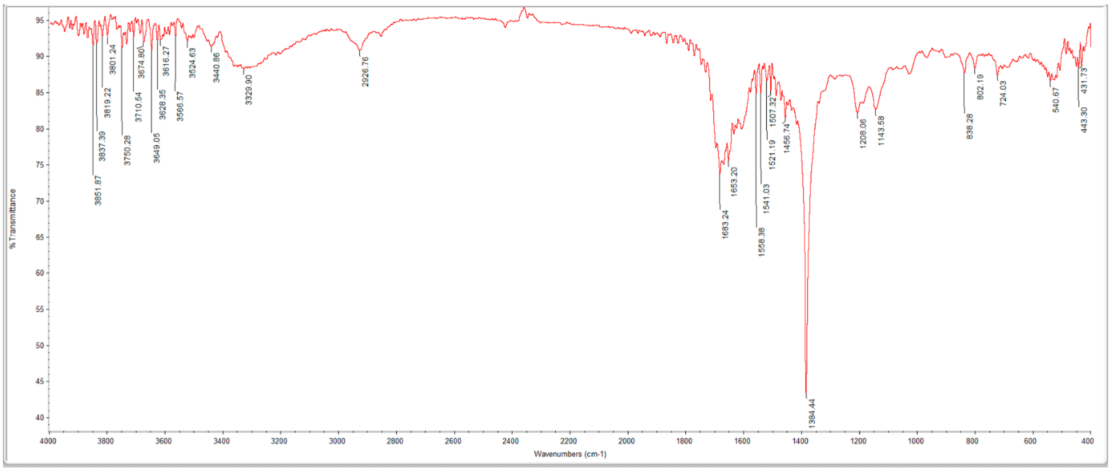

Figure S19. ^{1}H NMR (500 MHz, DMSO-d_6) spectrum of **3**.

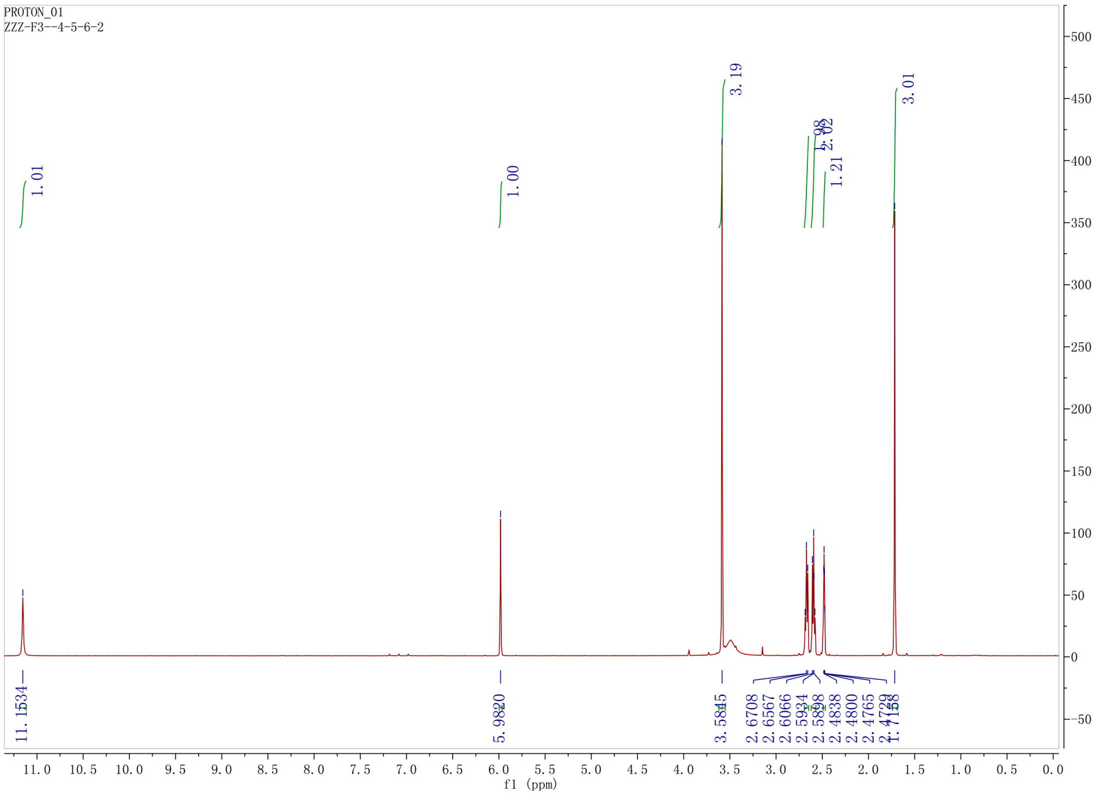

Figure S20. ^{13}C NMR (125 MHz, DMSO-d_6) spectrum of **3**.

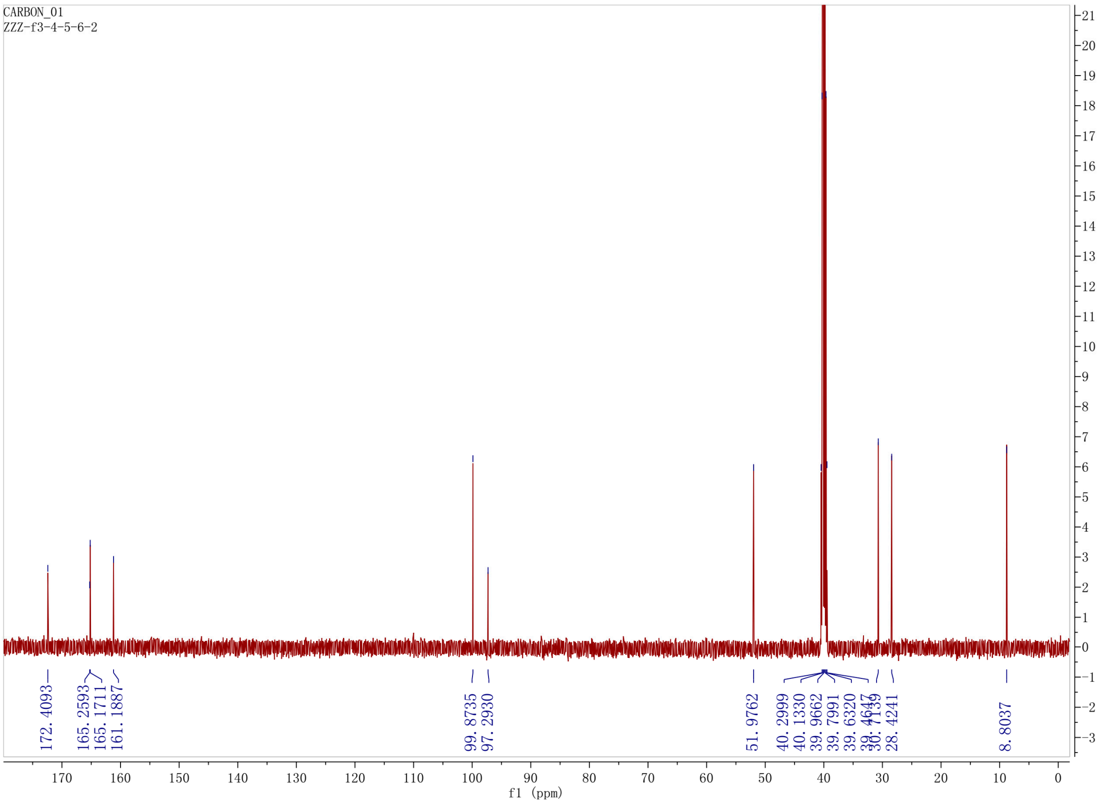

Figure S21. DEPT (125 MHz, DMSO-d_6) spectrum of **3**.

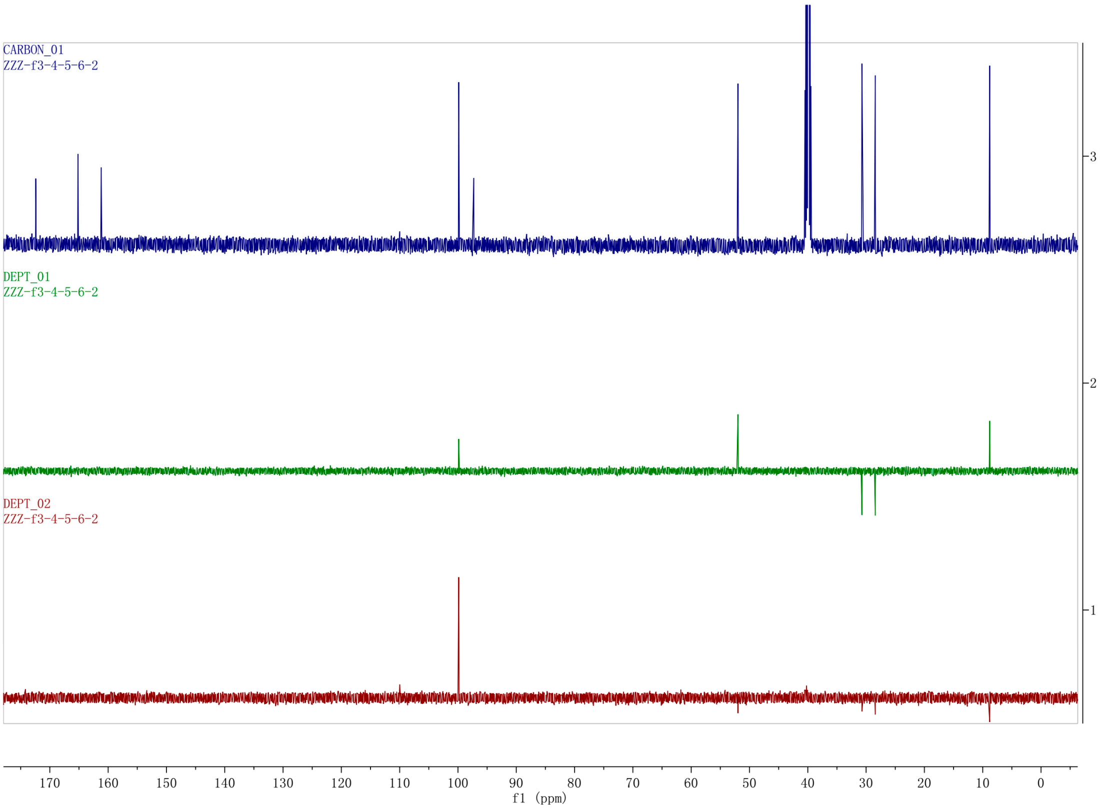

Figure S22. HSQC spectrum of **3** in DMSO-d_6.

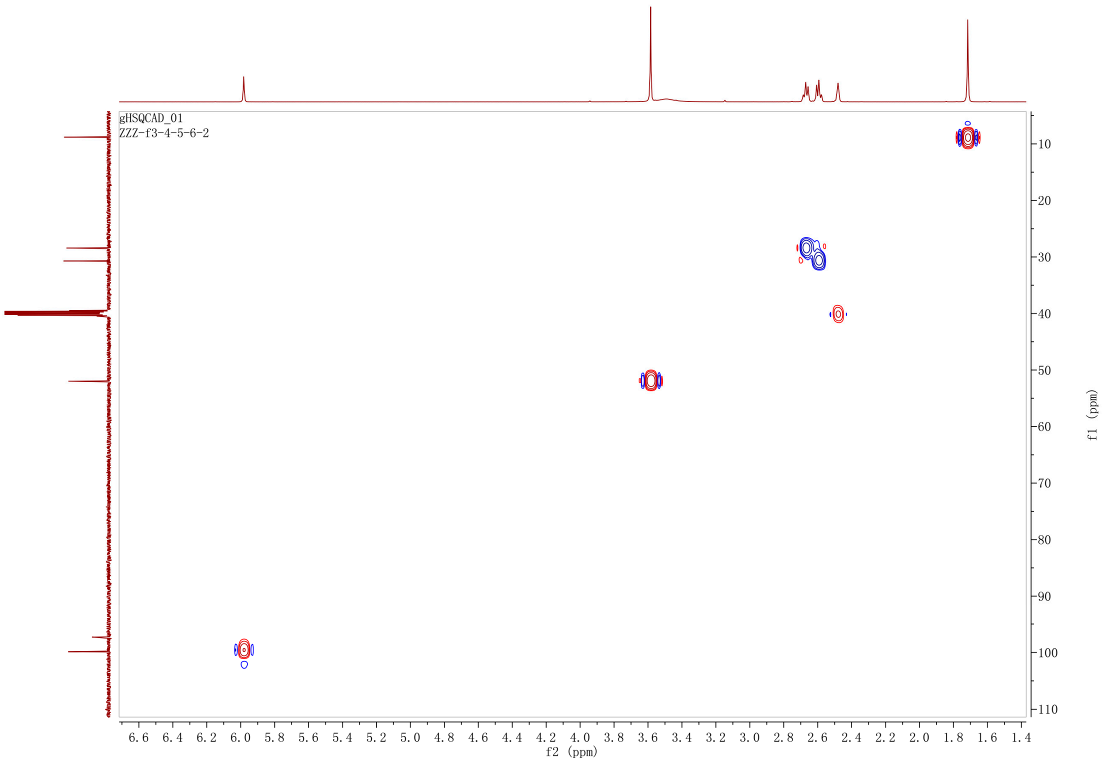

Figure S23. ^{1}H-^{1}H COSY spectrum of **3** in DMSO-d_6.

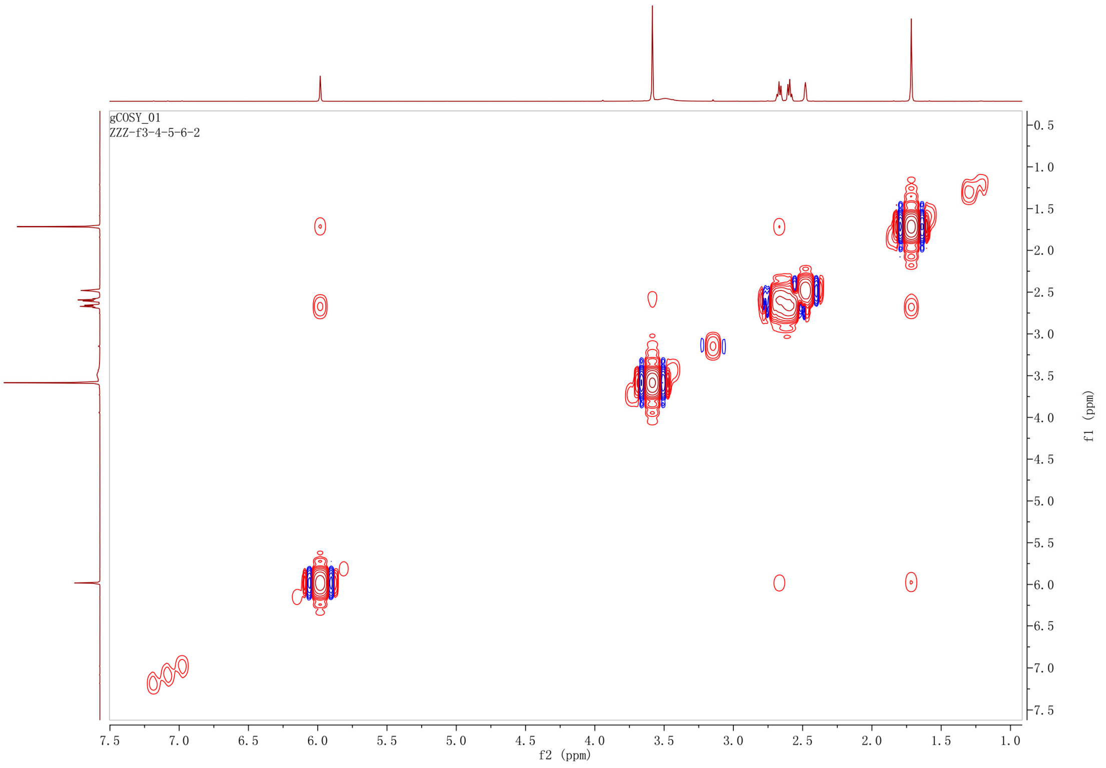

Figure S24. HMBC spectrum of **3** in DMSO-d_6.

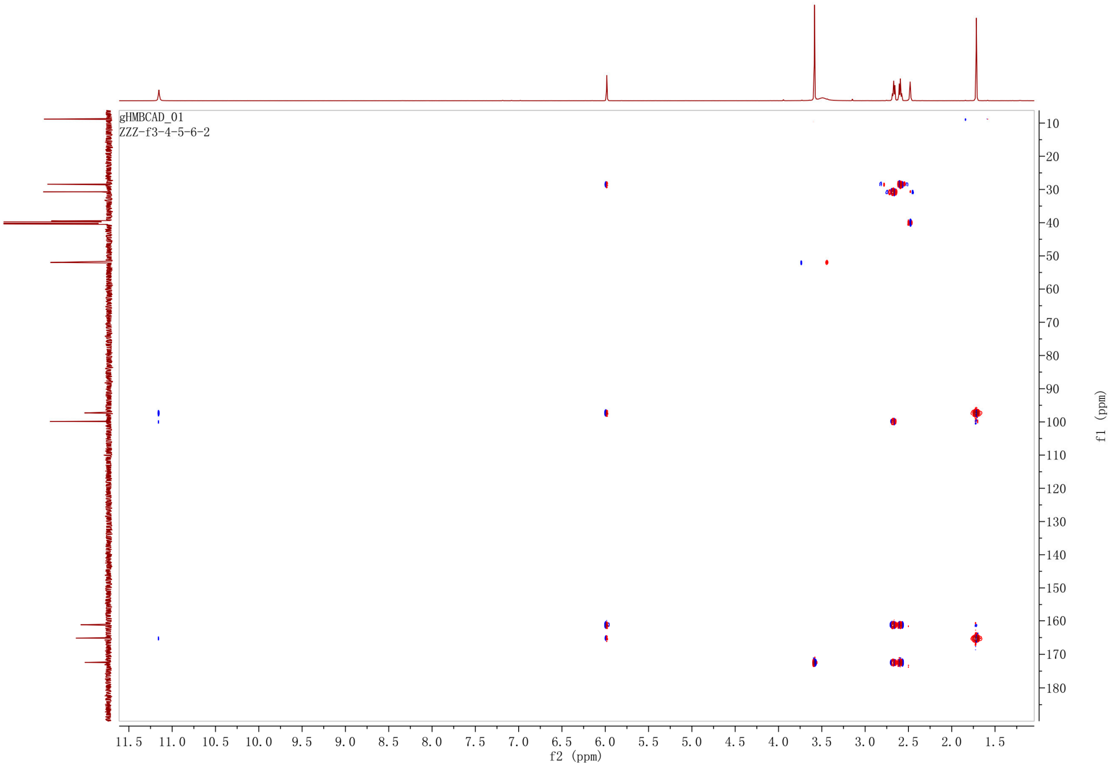

Figure S25. HRESIMS spectrum of **3**.

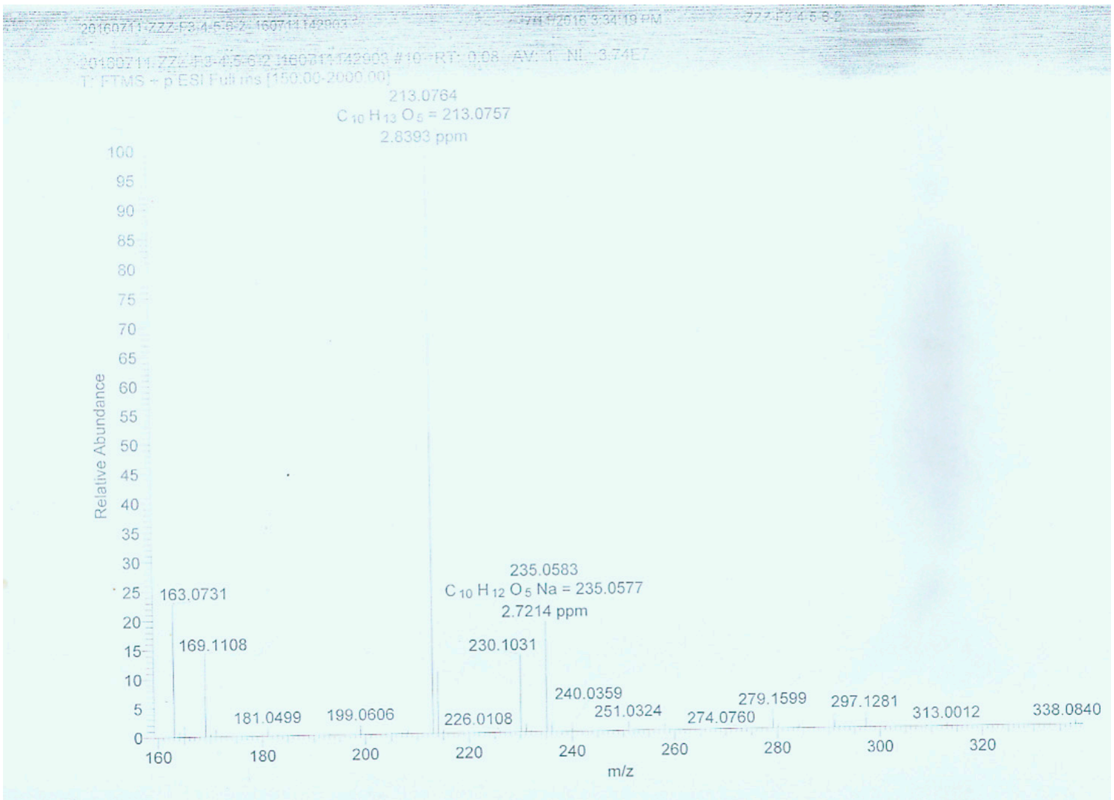

Figure S26. IR spectrum of **3**.

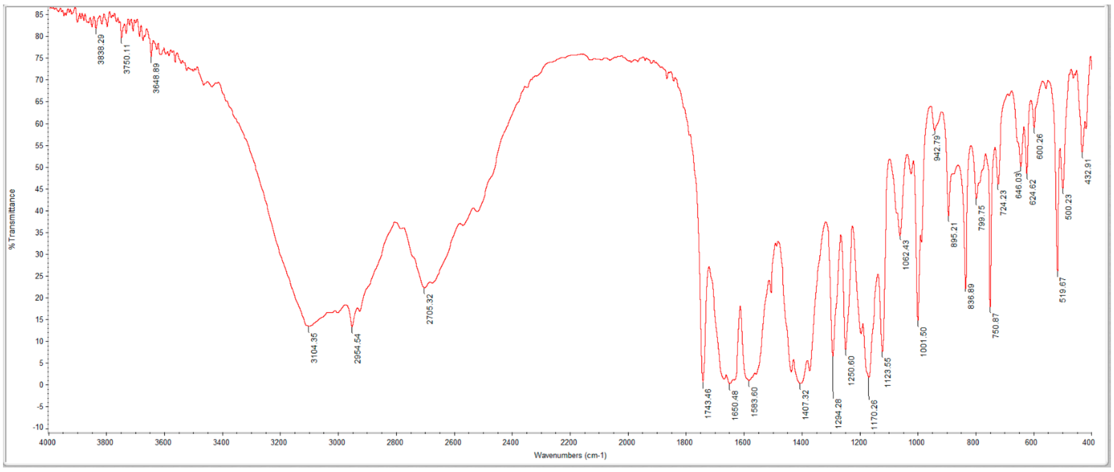

Figure S27. ECD calculation of **1a.**

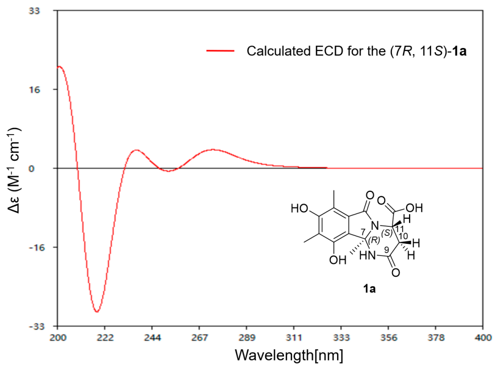

Figure S27-1. B3LYP/6-31+G(d) calculated spectrum of (7*R*, 11*S*)-**1a** (0.30 eV).

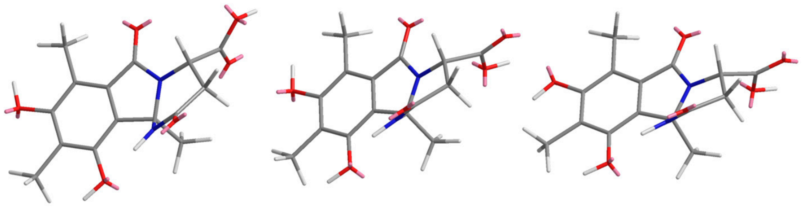

70.3% 25.2% 4.5%

Figure S27-2. DFT-optimized structures for low-energy conformers of (7*R*, 11*S*)-**1a** at B3LYP/6-31+g (d) level in methanol (CPCM) (conformer populations were calculated using the Gibbs free energy and Boltzmann population at 298 K thereof estimated).

Table S1. Cartesian coordinates of the low-energy reoptimized conformers of (7*R*, 11*S*)-**1a** calculated at B3LYP/6-31+G(d) level of theory with PCM solvent model for MeOH.

**Conformer A**	Standard Orientation (Ångstroms)

I	Atom	X	Y	Z
1	C	-3.39206	-0.72445	-0.14479
2	C	-3.32982	0.64183	0.19573
3	C	-2.06232	1.22366	0.37538
4	C	-0.91814	0.45115	0.21718
5	C	-1.0217	-0.89422	-0.13846
6	C	-2.25048	-1.53365	-0.3273
7	C	-2.38844	-2.99057	-0.70152
8	O	-4.65039	-1.24596	-0.30617
9	C	-4.5801	1.47331	0.35674
10	O	-1.89471	2.54981	0.68088
11	C	0.53326	0.85137	0.39787
12	N	1.22065	-0.39619	0.03646
13	C	0.36128	-1.41822	-0.30942
14	C	0.84281	1.28776	1.84239
15	O	0.73931	-2.52084	-0.7099
16	N	0.92597	1.88401	-0.57716
17	C	2.1875	2.01962	-1.05378
18	C	3.19433	0.99413	-0.54443
19	C	2.61532	-0.43427	-0.35427
20	C	3.47154	-1.22421	0.64087
21	O	3.14492	-1.54649	1.76353
22	O	4.67412	-1.5045	0.10215
23	O	2.51749	2.92929	-1.82227
24	H	-2.95287	-3.55224	0.05637
25	H	-2.90601	-3.11674	-1.66308
26	H	-1.40678	-3.45561	-0.79529
27	H	-4.59775	-2.18655	-0.53958
28	H	-4.61546	1.96715	1.33732
29	H	-4.64861	2.25412	-0.41373
30	H	-5.47355	0.85394	0.27277
31	H	-2.75168	3.00086	0.75152
32	H	1.90878	1.49135	1.97542
33	H	0.28336	2.19714	2.0766
34	H	0.54904	0.49812	2.53981
35	H	0.25379	2.61011	-0.80515
36	H	4.02971	0.96005	-1.24443
37	H	3.58774	1.38093	0.40393
38	H	2.67738	-0.96589	-1.31048
39	H	5.21258	-1.98135	0.76398

**Conformer B**	Standard Orientation (Ångstroms)

I	Atom	X	Y	Z
1	C	-3.37838	-0.7218	-0.14765
2	C	-3.31652	0.64624	0.18618
3	C	-2.04924	1.2291	0.36387
4	C	-0.90476	0.45651	0.2083
5	C	-1.00801	-0.89101	-0.13955
6	C	-2.23665	-1.53176	-0.3253
7	C	-2.37388	-2.99048	-0.69252
8	O	-4.63649	-1.24418	-0.30741
9	C	-4.56702	1.47831	0.34218
10	O	-1.88167	2.55643	0.66428
11	C	0.54627	0.85679	0.39284
12	N	1.23537	-0.39027	0.02706
13	C	0.37479	-1.41435	-0.30942
14	C	0.84697	1.28281	1.84304
15	O	0.75543	-2.5187	-0.70326
16	N	0.94291	1.89643	-0.57203
17	C	2.21133	2.04489	-1.02775
18	C	3.20847	1.00645	-0.52625
19	C	2.62192	-0.42426	-0.40338
20	C	3.54159	-1.27255	0.47822
21	O	4.60359	-1.71192	0.07919
22	O	3.08803	-1.44305	1.72935
23	O	2.55017	2.96911	-1.77417
24	H	-2.93654	-3.54917	0.06888
25	H	-2.89294	-3.12154	-1.65264
26	H	-1.39198	-3.4551	-0.78609
27	H	-4.58361	-2.18521	-0.53903
28	H	-4.60307	1.97703	1.32026
29	H	-4.63501	2.25525	-0.43221
30	H	-5.46033	0.85843	0.26066
31	H	-2.73847	3.00828	0.73184
32	H	1.91063	1.49174	1.98416
33	H	0.28032	2.18655	2.0818
34	H	0.55392	0.48565	2.53219
35	H	0.27275	2.62683	-0.79245
36	H	4.06076	0.99149	-1.20647
37	H	3.57823	1.35792	0.44468
38	H	2.65492	-0.8991	-1.3898
39	H	3.74867	-1.96309	2.22828

**Conformer C**	Standard Orientation (Ångstroms)

I	Atom	X	Y	Z
1	C	-3.36911	-0.72362	-0.14892
2	C	-3.31389	0.64575	0.1907
3	C	-2.04428	1.23407	0.35786
4	C	-0.90424	0.45335	0.20505
5	C	-1.00514	-0.8952	-0.14171
6	C	-2.23206	-1.53557	-0.32996
7	C	-2.38778	-2.98811	-0.70254
8	O	-4.5716	-1.35436	-0.33743
9	C	-4.59922	1.42576	0.3515
10	O	-1.84509	2.56044	0.64712
11	C	0.54699	0.85428	0.39268
12	N	1.23732	-0.39104	0.02449
13	C	0.37859	-1.41653	-0.31264
14	C	0.84593	1.27437	1.84531
15	O	0.76219	-2.51916	-0.70761
16	N	0.94598	1.89754	-0.56716
17	C	2.21612	2.04914	-1.01676
18	C	3.21173	1.00699	-0.51963
19	C	2.62423	-0.42376	-0.40447
20	C	3.54194	-1.27744	0.47395
21	O	4.60219	-1.71933	0.07309
22	O	3.08895	-1.44954	1.72521
23	O	2.55823	2.97907	-1.75475
24	H	-2.9719	-3.52471	0.05542
25	H	-2.93089	-3.09321	-1.65011
26	H	-1.41473	-3.46913	-0.8032
27	H	-5.31362	-0.74291	-0.20874
28	H	-5.2927	0.92169	1.03732
29	H	-4.45022	2.42123	0.77842
30	H	-5.11442	1.56936	-0.60772
31	H	-2.6835	3.04727	0.67859
32	H	1.9098	1.48076	1.9888
33	H	0.28065	2.17805	2.08709
34	H	0.55077	0.47473	2.5307
35	H	0.27789	2.63248	-0.77881
36	H	4.06485	0.99488	-1.19889
37	H	3.5807	1.35338	0.45343
38	H	2.65816	-0.89386	-1.39315
39	H	3.74814	-1.97335	2.22206

Figure S28. ECD calculation of **1b**.

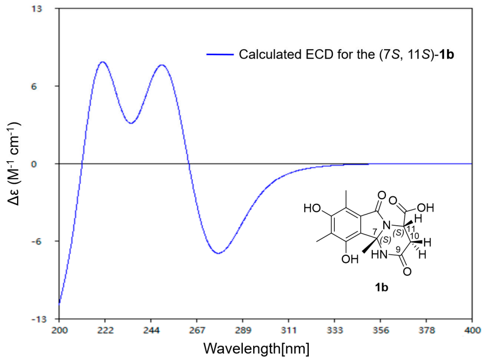

Figure S28-1. B3LYP/6-31+G(d) calculated spectrum of (7*S*, 11*S*)-**1b** (0.35 eV).

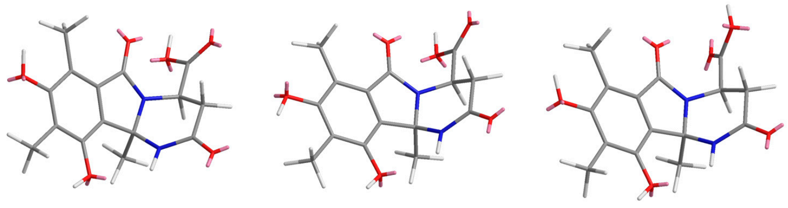

Figure S28-2. DFT-optimized structures for low-energy conformers of (7*S*, 11*S*)-**1b** at B3LYP/6-31+g (d) level in methanol (CPCM) (conformer populations were calculated using the Gibbs free energy and Boltzmann population at 298 K thereof estimated).

Table S2. Cartesian coordinates of the low-energy reoptimized conformers of (7*S*, 11*S*)-**1b** calculated at B3LYP/6-31+G(d) level of theory with PCM solvent model for MeOH.

**Conformer A**		Standard Orientation (Ångstroms)		
I	Atom	X	Y	Z
1	C	3.19567	0.97872	-0.25837
2	C	3.33372	-0.38635	0.06577
3	C	2.17245	-1.11742	0.37392
4	C	0.93464	-0.48603	0.35998
5	C	0.83867	0.86238	0.01385
6	C	1.95299	1.64502	-0.3044
7	C	1.87343	3.10832	-0.66994
8	O	4.35877	1.64756	-0.54391
9	C	4.68425	-1.06179	0.07625
10	O	2.19977	-2.45515	0.67235
11	C	-0.43789	-1.06302	0.66211
12	N	-1.28873	0.11891	0.50159

13	C	-0.60583	1.21631	0.00158
14	C	-0.55157	-1.66606	2.07034
15	O	-1.15726	2.24893	-0.37797
16	N	-0.83493	-2.06533	-0.35537
17	C	-1.90526	-2.0341	-1.19421
18	C	-2.85353	-0.84593	-1.08786
19	C	-2.70443	-0.11916	0.25174
20	C	-3.55761	1.14528	0.36165
21	O	-4.35089	1.51203	-0.48127
22	O	-3.41892	1.73065	1.56438
23	O	-2.10197	-2.93526	-2.01961
24	H	2.43058	3.73407	0.04154
25	H	2.28109	3.3002	-1.67261
26	H	0.83762	3.44855	-0.66854
27	H	4.17194	2.57602	-0.75613
28	H	4.90164	-1.51829	1.05152
29	H	4.74637	-1.85259	-0.68434
30	H	5.47979	-0.3462	-0.13311
31	H	3.10548	-2.80282	0.63308
32	H	0.09137	-2.54588	2.15292
33	H	-0.23795	-0.92909	2.81566
34	H	-1.58205	-1.97132	2.27806
35	H	-0.2343	-2.8808	-0.43694
36	H	-3.8713	-1.2173	-1.233
37	H	-2.63687	-0.15954	-1.91494
38	H	-3.06645	-0.76848	1.05868
39	H	-4.00545	2.51138	1.59846

**Conformer B**		Standard Orientation (Ångstroms)		
I	Atom	X	Y	Z
1	C	3.1878	0.9725	-0.25606
2	C	3.32867	-0.39573	0.0641
3	C	2.16398	-1.12574	0.37523
4	C	0.93182	-0.48176	0.36173
5	C	0.83675	0.86657	0.01353
6	C	1.95189	1.6462	-0.30356
7	C	1.89693	3.10786	-0.66875
8	O	4.2832	1.73985	-0.55732
9	C	4.70715	-1.01707	0.05759
10	O	2.15368	-2.4636	0.67647
11	C	-0.44167	-1.05576	0.6678

12	N	-1.29176	0.12543	0.4992
13	C	-0.60779	1.22129	-0.00277
14	C	-0.55838	-1.64754	2.08082
15	O	-1.15872	2.25181	-0.38786
16	N	-0.83915	-2.06555	-0.34205
17	C	-1.90697	-2.03821	-1.18375
18	C	-2.85481	-0.84898	-1.08611
19	C	-2.70714	-0.1136	0.24896
20	C	-3.55999	1.15185	0.34951
21	O	-4.35075	1.51452	-0.49758
22	O	-3.42504	1.74314	1.54981
23	O	-2.10247	-2.94373	-2.00489
24	H	2.49048	3.70719	0.03282
25	H	2.32175	3.27908	-1.66575
26	H	0.86972	3.47287	-0.66138
27	H	5.10154	1.22138	-0.50639
28	H	5.36436	-0.55124	0.804
29	H	4.70579	-2.08595	0.28678
30	H	5.18822	-0.92403	-0.92518
31	H	3.04583	-2.84246	0.64363
32	H	0.08236	-2.52789	2.17178
33	H	-0.24485	-0.90495	2.82062
34	H	-1.58997	-1.9487	2.28908
35	H	-0.23634	-2.87993	-0.41866
36	H	-3.8726	-1.22079	-1.23011
37	H	-2.63683	-0.16805	-1.91736
38	H	-3.07083	-0.75751	1.05951
39	H	-4.01093	2.52459	1.57788

**Conformer C**		Standard Orientation (Ångstroms)		
I	Atom	X	Y	Z
1	C	3.19622	0.99581	-0.25465
2	C	3.34395	-0.3724	0.05138
3	C	2.18894	-1.11383	0.35858
4	C	0.9478	-0.4887	0.36387
5	C	0.84232	0.86384	0.03642
6	C	1.9498	1.65609	-0.28176
7	C	1.85939	3.12345	-0.62786
8	O	4.35341	1.67428	-0.54145
9	C	4.69834	-1.04016	0.04436
10	O	2.22621	-2.45533	0.63803

11	C	-0.41989	-1.07707	0.66718
12	N	-1.27577	0.10502	0.54157
13	C	-0.60417	1.20929	0.03986
14	C	-0.51871	-1.71365	2.06115
15	O	-1.16636	2.23812	-0.33408
16	N	-0.8254	-2.05619	-0.37067
17	C	-1.891	-1.99568	-1.21426
18	C	-2.83545	-0.80578	-1.07795
19	C	-2.6913	-0.13305	0.29274
20	C	-3.52471	1.12829	0.54523
21	O	-3.528	1.72589	1.60245
22	O	-4.3324	1.42987	-0.48458
23	O	-2.08904	-2.87285	-2.06467
24	H	2.42145	3.74293	0.08521
25	H	2.25497	3.32982	-1.63251
26	H	0.82214	3.45877	-0.61072
27	H	4.16	2.60451	-0.73958
28	H	4.92095	-1.51477	1.00973
29	H	4.76326	-1.81513	-0.73214
30	H	5.4892	-0.31586	-0.15248
31	H	3.13318	-2.79753	0.58357
32	H	0.12486	-2.59508	2.11697
33	H	-0.19755	-0.99413	2.8201
34	H	-1.54708	-2.02336	2.2725
35	H	-0.22774	-2.87139	-0.47373
36	H	-3.85231	-1.16919	-1.24732
37	H	-2.60361	-0.09489	-1.87941
38	H	-3.0378	-0.82836	1.06687
39	H	-4.87553	2.20314	-0.23481
